# Prostate cancer exploits BRD9-driven metabolic reprogramming to shape the aggressive phenotype

**DOI:** 10.1038/s41419-025-07561-9

**Published:** 2025-04-22

**Authors:** Ye Lv, Xinkai Mo, Ruojia Zhang, Yu Peng, Tingting Feng, Yuang Zhang, Guanhua Song, Luna Ge, Yu Liu, Guiwen Yang, Lin Wang

**Affiliations:** 1 Shandong Provincial Key Laboratory of Animal Resistance Biology, College of Life Sciences, Shandong Normal University, Jinan, Shandong China; 2 Department of Clinical Laboratory, Shandong Cancer Hospital and Institute, Shandong First Medical University & Shandong Academy of Medical Sciences, Jinan, Shandong China; 3 Biomedical Sciences College & Shandong Medicinal Biotechnology Centre, NHC Key Laboratory of Biotechnology Drugs, Key Lab for Rare & Uncommon Diseases of Shandong Province, Shandong First Medical University & Shandong Academy of Medical Sciences, Jinan, Shandong China; 4 The Key Laboratory of Experimental Teratology, Ministry of Education and Department of Pathology, School of Basic Medical Sciences, Cheeloo College of Medicine, Shandong University, Jinan, Shandong China; 5 Department of Rheumatology and Autoimmunology, The First Affiliated Hospital of Shandong First Medical University, Jinan, Shandong China; 6 Department of Immunology, School of Clinical and Basic Medical Sciences, Shandong First Medical University & Shandong Academy of Medical Sciences, Jinan, Shandong China

**Keywords:** Oncogenes, Cell growth, Cancer metabolism

## Abstract

The aggressive phenotype of prostate cancer (PCa) requires adaptation to androgen deprivation (AD) to progress into castration-resistant PCa (CRPC), including adaptation to AD-induced oxidative stress. However, our understanding of the oncogenes that maintain the redox balance during CRPC progression is limited. Here, we identified Bromodomain-containing protein 9 (BRD9) as a metabolic checkpoint for reprogramming cell metabolism to support tumor growth and impart a castration-resistant phenotype under metabolic and oxidative stress. Following oxidation, BRD9 recruited the nuclear transcription factor-Y A-subunit (NFYA) to induce glycogen phosphorylase L (PYGL) expression, which directed glucose utilization through the pentose phosphate pathway, generating NADPH, and promoting clearance of reactive oxygen species (ROS), thus maintaining redox balance. By disturbing redox homeostasis, BRD9 inhibition exerted oxidative pressure on PCa cells, sensitizing them to radiotherapy. This work identified BRD9 as a novel component in antioxidant reprogramming and indicates BRD9 targeting as a promising treatment strategy for PCa therapy.

## Introduction

Androgen deprivation therapy is still the mainstay of treatment for metastatic prostate cancer (PCa); however, resistance to this therapy ultimately develops in the form of castration-resistant PCa (CRPC) [1, 2]. Oxidative stress is closely related to PCa progression [3], which is characterized by the excessive production and accumulation of reactive oxygen species (ROS) [4]. Most cellular ROS are produced by cellular respiration in mitochondria [5], and previous studies showed that pharmaceutical and genetic inhibition of the androgen receptor (AR) could induce ROS production in both “hormone-sensitive” PCa cell lines and primary patient-derived PCa explants [6–8]. More significantly, despite the tumor-promoting effects of ROS signaling, CRPC cells also exploit antioxidant systems to efficiently clear ROS to reduce tumor-constricting effects [9]. As CRPC cells with an imbalance of ROS are more susceptible to oxidative stress-induced DNA damage and anti-tumor responses [10], identifying novel elements in antioxidant systems could be helpful for providing alternative treatment strategies.

The switch/sucrose non-fermentable (SWI/SNF) complex regulates gene expression by altering the chromatin structure via integrating various pathways that include both oncogenes and tumor suppressors [11–16]. The SWI/SNF complex has tumor-promoting functions in PCa, including the lethal castration-resistant neuroendocrine PCa [17]. Inhibition of the SWI/SNF ATPase subunits, SMARCA2 and SMARCA4, could potently inhibit tumor growth of PCa and synergize with the AR antagonist enzalutamide for better tumor control, even inducing disease remission in CRPC models, suggesting that SWI/SNF subunits serve as potential therapeutic targets in PCa [18–20]. In addition, the bromodomain-containing protein 9 (BRD9), as a component of the newly identified SWI/SNF complex, which has the potential to be a diagnostic and prognostic biomarker in PCa [21]. Bromodomain-containing proteins recognize acetylated lysine residues on histones and epigenetically regulate transcription, chromatin remodeling, and histone modification. BRD9 blockade could also suppress AR-target gene induction, even in the castration-resistant state, indicating the possibility that BRD9 could serve as a treatment target in therapy-resistant PCa, even though the exact role of BRD9 in PCa progression requires further clarification [22].

The development of CRPC is characterized by the reprogramming of global cellular metabolic pathways, including the deregulation of mitochondrial oxidative phosphorylation (OXPHOS), the aberrant utilization of glycolysis, and the “Warburg effect” [23–26]. During glycogenolysis, glycogen phosphorylase (PYG) isozymes break down glycogen into glucose-1-phosphate (G1P) for further catabolism via glycolysis [27]. As an alternative metabolic pathway downstream of glycolysis upon glucose breakdown, the pentose phosphate pathway (PPP) provides precursors for nucleotide biosynthesis and generates NADPH [28]. Depending on the cell requirements, NADPH can be directed for anabolic reactions or for maintaining cellular redox homeostasis in a cell-state-dependent way [29]. Therefore, PPP contributes to the generation of ROS scavengers and can serve as an important component in antioxidant reprogramming [28].

In this study, we uncovered a novel role for BRD9 in maintaining redox homeostasis that promotes CRPC. Specifically, PCa cells with BRD9-overexpression survived androgen deprivation-induced oxidative stress due to the antioxidant activity of BRD9 antioxidant. Mechanistic analysis linked BRD9 and factor-Y A-subunit (NFYA) to PYGL-initiated glucose utilization that enables the glycolysis-PPP axis to keep a redox balance in favor of mitochondrial respiration. Notably, pharmacological and genetic suppression of BRD9 results in defective and inactivated glycolysis-PPP, impaired antioxidant activity, defective mitochondrial OXPHOS, pronounced DNA damage, and PCa regression. Our results uncover an important pathophysiological role of BRD9 in exploiting glucose utilization, which constitutes an adaptive survival mechanism that permits PCa cells to survive metabolic stress. These findings have potential implications for new metabolism-based anticancer therapies.

## Materials and methods

### Cell culture

Human PCa LNCaP and C4-2B cells and mouse PCa RM-1 cells were purchased from the American Type Culture Collection (ATCC; Manassas, VA, USA) and cultured following ATCC’s instructions. Mycoplasma PCR testing of these cells was performed and validated by short tandem repeat assays. The cumulative culture length of the cells between thawing and use in this study was less than 15 passages. For the androgen deprivation, LNCaP cells were hormone-starved in the Phenol Red-free medium containing 10% charcoal-stripped fetal bovine serum (CSS; Hyclone, Logan, UT, USA). N-acetyl-L-cysteine (NAC), dihydrotestosterone (DHT), and DL-dithiothreitol (DTT) were purchased from MedChemExpress. BRD9 inhibitor (I-BRD9) was purchased from Selleck. CP-320626 was purchased from Topscience.

### Immunohistochemistry

Immunohistochemistry (IHC) was carried out as described previously [30]. Antigen retrieving was performed in Tris (pH 6.0) in a pressure cooker for 10 min. The tissue slides were incubated with the indicated primary antibodies overnight at 4 °C. Primary antibody used in this study was anti-Ki67 (ZA-0502, Zsbio). IHC intensity was assessed as described before [31].

### Immunofluorescence

Immunofluorescence (IF) was carried out as described previously [32]. Pretreated PCa cells were seeded on glass coverslips in 24-well plates (2 × 10^4^ cells/well), fixed in 4% paraformaldehyde, permeated by 0.5% Triton X-100, and blocked with 5% goat serum sealant then incubated with primary antibodies overnight at 4 °C, followed by incubation with secondary antibodies. Images were processed under a confocal microscope (FV3000, OLYMPUS, Tokyo, Japan).

### RNA isolation and real-time qPCR analysis

Total RNA was extracted using TRIzol reagent (Vazyme) (Biotech, R401-01 reagent kit) and reverse transcribed using the ReverTra Ace qPCR RT kit (Toyobo, PCR-311) according to the manufacturer’s protocol. Real-time qPCR was performed using SYBR Green mix (Toyobo, QPK-201). The primer sequences used are listed in Supplementary Table 1.

### Western blotting and immunoprecipitation

Western blotting and immunoprecipitation analyses were carried out as described previously [33]. Antibody information is summarized in Supplementary Table 2.

### Transient transfection and viral transduction

Human BRD9 and PYGL plasmids were purchased from Sangon Biotech (Shanghai, China). All siRNAs were purchased from Genepharma (Guangzhou, China), and the sequences of siRNAs are listed in Supplementary Table 3. Lipofectamine 3000 (Invitrogen, Carlsbad, CA) was used for transfection following the manufacturer’s instructions. To avoid off-target effects, we cotransfected two siRNAs for better interference efficiency. Human Lenti-BRD9-EGFP and its control Lenti-EGFP were obtained from Genecopoeia. To acquire a stable cell line, single-cell clonal isolates were selected by puromycin.

### Cell proliferation and colony formation

To determine the effects of BRD9 on proliferation of PCa cells, cellular proliferation was measured by CCK-8 (Promega, Madison, WI, USA), the BeyoClick™ EdU-647 (EdU) assay (Beyotime, Shanghai, China), and clonal formation assays. For CCK-8 assay, the absorbance of each well was measured by a microplate reader at an excitation wavelength of 450 nm.

### Tumor models

Male nude mice aged 4–6 weeks were purchased from Huafukang Biotechnology (Beijing, China), maintained under pathogen-free conditions, and handled according to the guidelines for animal experiments. The CRPC model was used as described previously [30]. Stable knockdown or overexpression of BRD9 in 1 × 10^7^ LNCaP cells or 1 × 10^7^ C4-2B cells and corresponding controls were suspended in 100 μl of PBS containing 50% Matrigel. The mice were injected subcutaneously with the cells, and tumor volume was measured every 3 days. Tumor volume was calculated as volume = length × width^2^/2. Mice-bearing tumors were castrated when the tumor size was ~200 mm^3^. Mice were then randomized and treated once daily (*n* = 5/group) with CP-320626 (100 mg/kg, i.v.) or vehicle control (PBS). For combination treatment with enzalutamide and I-BRD9, mice were randomly selected to receive enzalutamide (10 mg/kg, p.o.) plus I-BRD9 (20 mg/kg, p.o.) or PBS (p.o.) once daily (*n* = 5/ group).

C57BL/6 mice were purchased from Huafukang Biotechnology (Beijing, China). 1 × 10^6^ mouse PCa RM-1 cells were injected subcutaneously into the right posterior abdomen of the mice. When the subcutaneous tumor volume reached 200 mm^3^, the mice were randomly divided into 4 groups (the mice with tumors were randomly assigned), with 5 mice in each group, the group requiring RT received local RT of 15 Gy with the body partially covered with a lead block. Tumor volume was measured every 3 days. Tumor volume was calculated as volume = length × width^2^/2. Mice were euthanized when tumors exceeded 2 cm in diameter, and tumors were isolated, weighed, photographed, and fixed for immunohistochemical analysis. The experimental protocol was performed in accordance with the requirements of the Animal Care and Use Ethics Committee of Shandong First Medical University (D20230117017).

### RNA sequencing (RNA‑seq) and bioinformatics analysis

We performed RNA-Seq analysis (Lianchuan, Shanghai, China) to compare the mRNA expression profiles between control (siNC) and BRD9 knockdown (siBRD9) LNCaP-AI cells that became androgen-independent after long-term deprivation of androgen in LNCaP. Biological enrichment analysis of the expressed genes was performed using Gene Set Enrichment Analysis (GSEA) (http://software.broadinstitute.org/gsea/index.jsp). Datasets of GSE2443, GSE8702, GSE3325, GSE35988, and GSE68882 were downloaded from the Gene Expression Omnibus (GEO) database (http://www.ncbi.nlm.nih.gov/geo). GEPIA (http://gepia.cancer-pku.cn/detail.php) was used to analyze the relationship between BRD9 expression and disease-free survival (DFS) of PCa cases.

### Statistical analysis

GraphPad Prism 8 software was used for statistical analysis. Two-sided Student’s *t* test was used to calculate statistical significance between two groups. ANOVA was used for comparison among multiple groups. All results are presented as mean ± SD. Tumor growth was analyzed by analysis of variance. Kaplan–Meier curves and log-rank test were used to compare DFS in different patient groups. *P* values were considered to be significant as follows: *P < 0.05, **P < 0.01, ***P < 0.001.

## Results

### BRD9 is sufficient to induce the CRPC-like phenotype of PCa cells

**Fig. 1:**
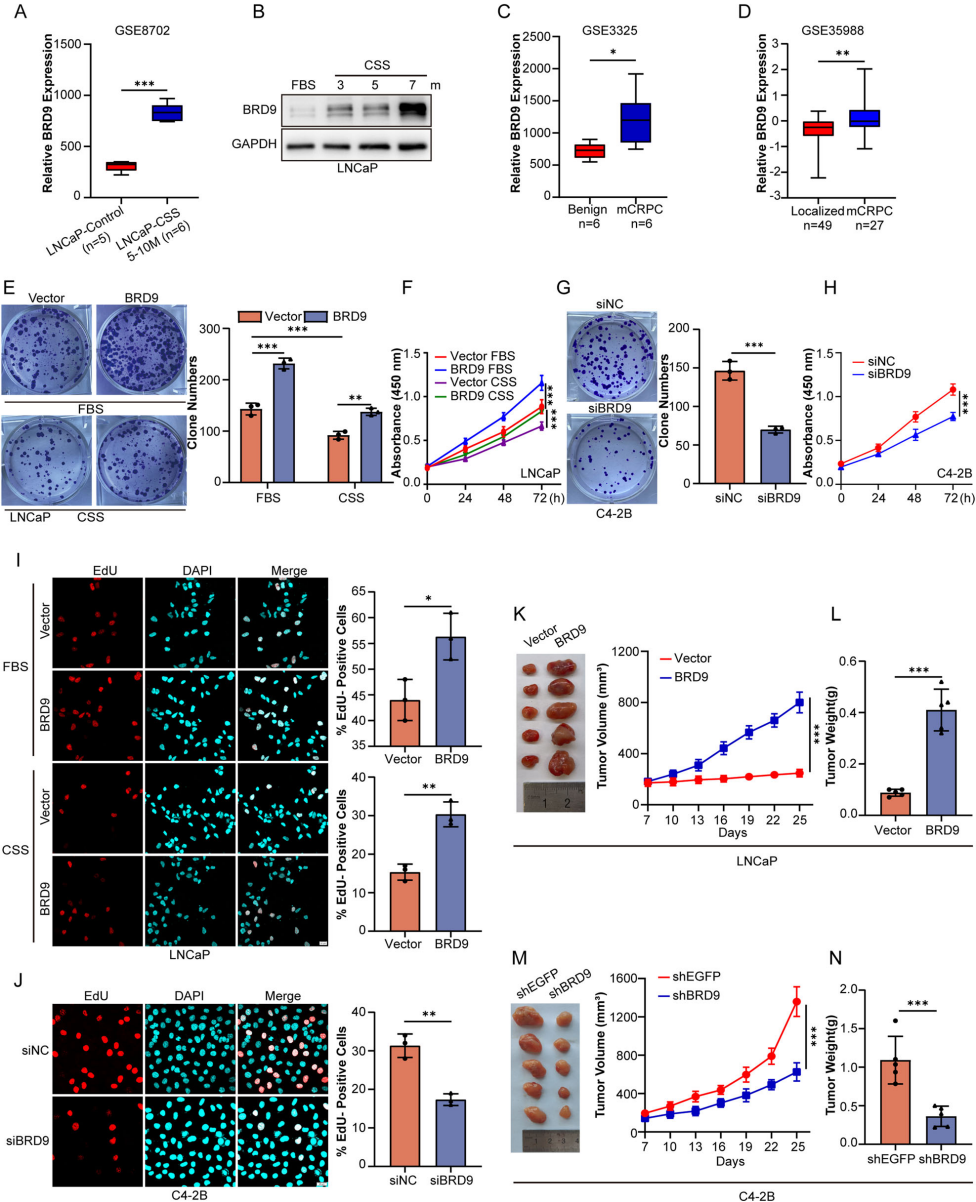
Upregulation of BRD9 expression in CRPC and PCa cell growth promotion by BRD9 in vitro and in vivo. **A** BRD9 expression in LNCaP cells with different times of androgen deprivation, from public datasets (GSE8702). **B** Protein levels of BRD9 in LNCaP cells with prolonged androgen-deprived treatment (3, 5, and 7 months) determined by western blotting. GAPDH (glyceraldehyde-3-phosphate dehydrogenase) was used as a loading control. FBS fatal bovine serum, CSS charcoal-stripped serum, m months. **C**, **D** Comparison of BRD9 expression levels in various prostate tissues from different datasets (GSE3325 and GSE35988). **E**, **F** Colony formation and CCK-8 assays in LNCaP cells with BRD9 overexpression with or without androgen deprivation (*n* = 3). **G**, **H** Colony formation and CCK-8 assays in C4-2B cells with or without BRD9 silencing (siBRD9 or siNC, respectively). Representative images of colony formation are shown in the left panel and quantitative analysis is shown in the right panel. FBS fatal bovine serum, CSS charcoal-stripped serum. **I**, **J** EdU assays in PCa cells with BRD9 overexpression (BRD9 vs Vector, I) or knockdown (siBRD9 vs siNC, J) (*n* = 3). Representative images are shown in the left panels and quantitative analyses are shown in the right panels. Scale bars, 20 μm. Tumor volumes in nude mice with castration as the indicated treatment. LNCaP cells with stable overexpression (**K**, **L**) of BRD9 or C4-2B cells with stable knockdown of BRD9 (**M**, **N**), as well as their parental controls (vector or shEGFP), were subcutaneously injected into nude mice (*n* = 5/group). Tumor volume was measured every 3 days. Tumors were isolated from mice, weighed at the endpoint (**K**, **L**). EGFP, enhanced green fluorescent protein. Two-tailed unpaired *t*-test, one-way and two-way analysis of variance (ANOVA). Error bars represent SD; *P < 0.05, **P < 0.01, *** *P* < 0.001.

Comparative expression analysis using public datasets (GSE8702, Fig. 1A) and in our in vitro experiment (Fig. 1B) showed that BRD9 increased steadily in androgen-dependent PCa cell line LNCaP when the time periods were prolonged under androgen-deprived conditions. Further analysis from two independent datasets (GSE3325 and GSE35988) demonstrated that BRD9 expression progressively and significantly increased from benign and primary localized PCa to metastatic CRPC (mCRPC) tissues (Fig. 1C, D), suggesting an increasing requirement for BRD9 during progression to CPRC.

To determine the functional role of BRD9 in the proliferation capacity of the established CRPC cells, colony formation (Fig. 1E), Cell Counting Kit-8 (CCK-8) (Fig. 1F) and EdU (Fig. 1I) assays were conducted, and the results confirmed that BRD9 overexpression promoted the growth of PCa cells under both normal and hormone-free conditions. In contrast, the suppression of BRD9 expression significantly inhibited cell proliferation (Fig. 1G, H, J).

In vivo, the tumor volumes increased more rapidly in LNCaP-BRD9-derived tumors when compared to the parental controls at 3 weeks post-castration. At the time of euthanasia, the tumor volume was larger in the LNCaP-BRD9 group compared with control group (Fig. 1K, L). In contrast, C4-2B cells stably expressing BRD9 shRNA displayed reduced growth in castrated nude mice (Fig. 1M, N). Importantly, BRD9 inhibitor (I-BRD9) synergized with enzalutamide to inhibit cell proliferation (Fig. S1A, B) and delay tumor growth (Fig. S1C–F). These results suggest that, in addition to its established role in regulating AR signaling and PCa progression, BRD9 may behave as a modulator for PCa cells to adapt to androgen deprivation and therefore induce the CRPC phenotype.

### BRD9 promotes PCa progression via maintaining redox homeostasis

**Fig. 2:**
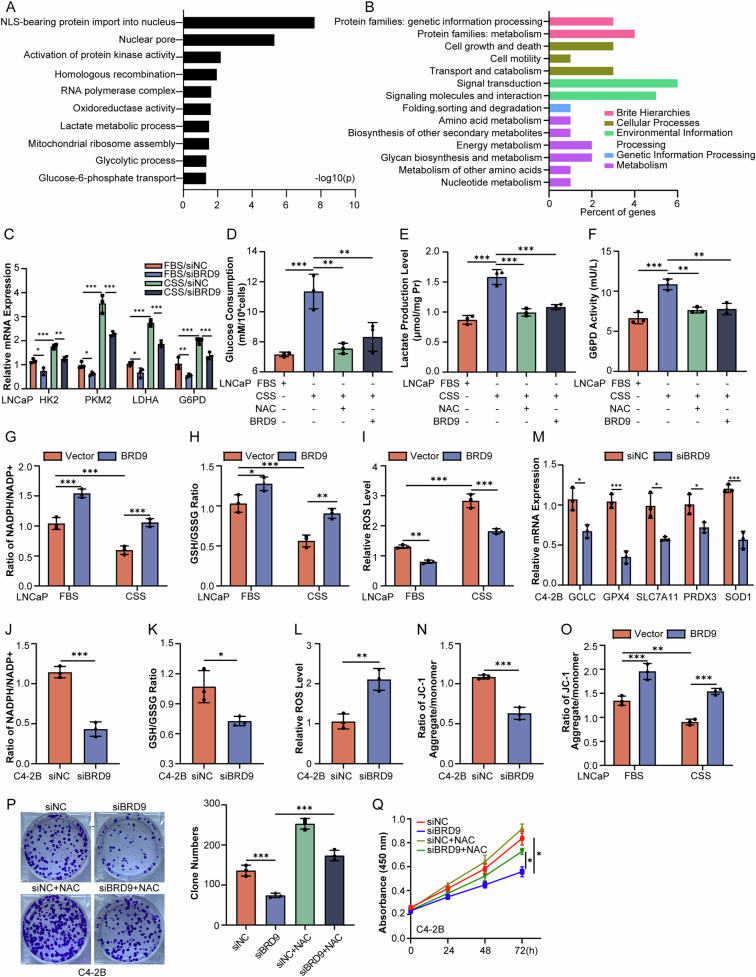
BRD9 affects REDOX balance in PCa cells. **A** DEGs in RNA-seq from LNCaP-AI shBRD9 cells were subjected to GO enrichment analysis. **B** Functional enrichment of BRD9 target genes in C4-2B cells from ChIP analysis. **C** Relative expression levels of HK2, PKM, LDHA, and G6PD in LNCaP cells transfected with siBRD9 or siNC 24 h with or without androgen deprivation (*n* = 3). **D**–**F** Levels of glucose consumption, lactate production, and G6PD activity in LNCaP cells (*n* = 3). **G**–**I** NADPH/NADP^+^ ratio, GSH/GSSG ratio, and ROS level in LNCaP cells transfected with BRD9 overexpression for 24 h with or without androgen deprivation. **J**–**L** NADPH/NADP^+^ ratio, GSH/GSSG ratio, and ROS level in C4-2B cells transfected with siBRD9 or siNC for 24 h (*n* = 3). **M** mRNA levels of antioxidant-related molecules in C4-2B cells (*n* = 3). **N**, **O** Membrane potential in C4-2B and LNCaP cells with BRD9 knockdown or overexpression with or without androgen deprivation (*n* = 3). **P**, **Q** Colony formation and CCK-8 assays in C4-2B cells transfected with siNC or siBRD9 in the presence of ROS scavenger NAC (5 nM) (*n* = 3). Representative images are shown in the left panel and quantitative analysis is shown in the right panel (**P**). Two-tailed unpaired *t*-test, one-way and two-way analysis of variance (ANOVA). Error bars represent SD; *P < 0.05, **P < 0.01, *** *P* < 0.001.

To mechanistically define the function of BRD9 in CRPC progression, we analyzed the RNA-Seq data from the BRD9 knockdown experiment in androgen-independent LNCaP-AI cells derived from long-term androgen-deprived LNCaP cells. We identified 233 differentially expressed genes (DEGs), i.e. genes that were significantly differentially regulated (DEGs) (*P* < 0.05, |log_2_FC| > 1) in BRD9 knockdown versus control cells. We also used Gene Ontology (GO) analyses to identify cellular functions associated with BRD9 and found that, besides the known functions such as homologous recombination, BRD9 expression was also predicted to impact distinct markers and pathways associated with oxidoreductase activity, lactate metabolic processes and glycolytic metabolism (Fig. 2A). A ChIP-seq experiment was also performed, and subsequent GO analysis showed that the potential targets for BRD9 were closely related to metabolism, especially glycan biosynthesis and metabolism (Fig. 2B). Additionally, BRD9 was positively associated with the glycolytic molecules HK2, PKM, LDHA and G6PD in PCa tissues of GEPIA database (Fig. S2). This association held true when the expression levels of these glycolytic genes were analyzed in LNCaP cells with BRD9 knockdown (Fig. 2C). Notably, in response to androgen deprivation, PCa cells were characterized by the enhancement of intracellular glucose consumption (Fig. 2D), lactate production (Fig. 2E) and G6PD enzyme activity (Fig. 2F), whereas such induction was abolished when the ROS scavenger N-acetyl cysteine (NAC) was used. The abolishment effect was also observed when BRD9 was overexpressed in LNCaP cells (Fig. 2D–F). Detailed analysis showed that androgen deprivation led to the decreased ratios of NADPH/NADP^+^ (Fig. 2G) and GSH/GSSG (Fig. 2H) but the increased production of ROS in LNCaP cells (Fig. 2I), whereas BRD9 overexpression could restore the ratios of NADPH/NADP^+^ and GSH/GSSG, as well as ROS production almost to their basal levels (Fig. 2G–I). In contrast, the ratios of NADPH/NADP^+^ and GSH/GSSG decreased whereas the production of ROS increased when BRD9 was silenced in C4-2B cells (Fig. 2J–L), which was also accompanied by the reduction of antioxidant factors (Fig. 2M). Additionally, BRD9 inhibition plus enzalutamide could synergistically provoke ROS production when compared to the ones alone (Fig. S1G).

As mitochondrial metabolism serves as the main source of ROS and its dysfunction is also characterized by the reduction in mitochondrial membrane potential and the disability to produce ATP [34, 35], we also examined the effects of BRD9 on mitochondrial activity. As shown in (Fig. S3A), BRD9 overexpression could reverse the decline of oxygen consumption rate (OCR) in LNCaP cells resulting from enzalutamide treatment or androgen deprivation, while such a decline became more obvious when BRD9 was knocked down in C4-2B cells (Fig. S3B), and when BRD9 was knocked down in C4-2B cells the mitochondria exhibit irregular morphology, with the cristae becoming loose (Fig. S3C). The same trend was observed when the mitochondrial membrane potential was examined (Fig. 2N, O). Notably, the addition of NAC could effectively restore the proliferation defect conferred by BRD9 knockdown in C4-2B cells (Fig. 2P, Q). Overall, these findings suggest that the elevated levels of BRD9 in CRPC cells contribute to alleviation of oxidative stress in favor of balancing cellular redox homeostasis.

### PYGL serves as a direct target for BRD9 to promote CRPC progression

**Fig. 3:**
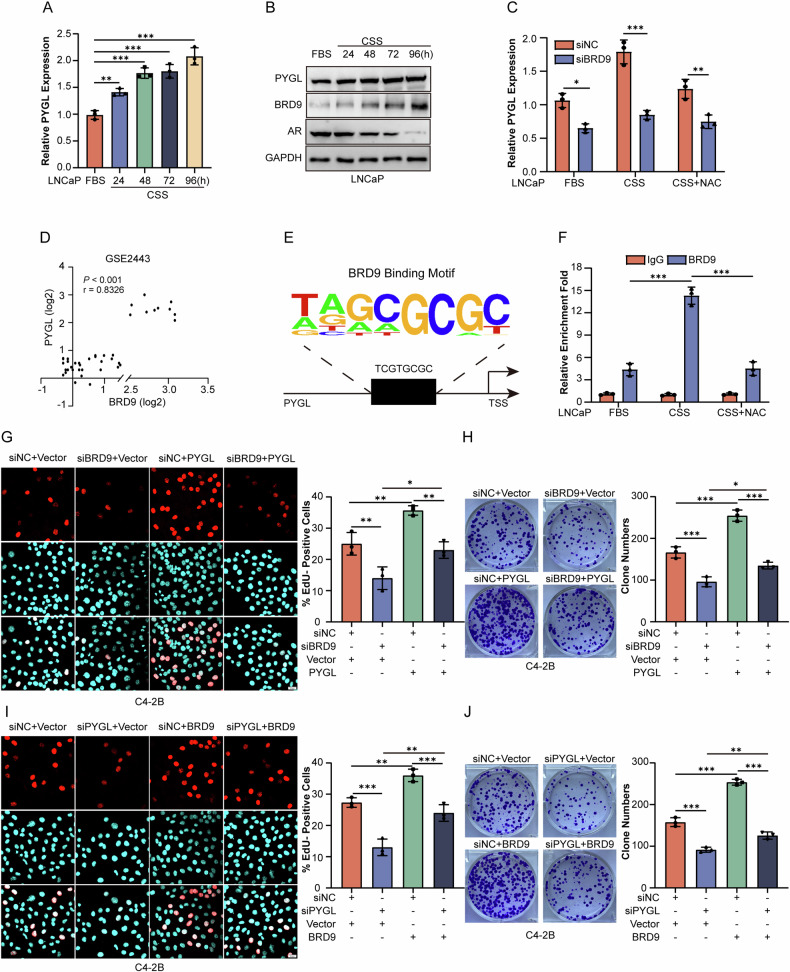
BRD9 promotes PCa cell growth through PYGL in PCa cells. **A**, **B** The mRNA and protein levels of PYGL in LNCaP cells with short-period androgen-deprivation treatment (24, 48, 72, and 96 h) determined by qRT-PCR and western blotting (*n* = 3). FBS fatal bovine serum, CSS charcoal-stripped serum, h hours. **C** Expression levels of PYGL mRNA in LNCaP cells transfected with siBRD9 or siNC 24 h post with or without androgen deprivation plus NAC treatment determined by qRT-PCR analyses (*n* = 3). **D** Correlation between the relative levels of BRD9 and PYGL mRNA transcripts in PCa tissues from the GSE2443 database. **E** The prediction of the binding site of BRD9 on the PYGL promoter based on ChIP-seq analysis. **F** ChIP analysis of the BRD9 enrichment on the PYGL promoter in LNCaP cells. Results show the relative enrichment with the anti-BRD9 antibody versus the IgG control. **G**, **H** EdU and colony formation assays in C4-2B cells with transfection of siBRD9 for 24 h and then the PYGL overexpression plasmid for another 24 h (*n* = 3). Scale bars, 20 μm. **I**, **J** EdU and colony formation assays in C4-2B cells with transfection of siPYGL for 24 h and then the BRD9 overexpression plasmid for another 24 h (*n* = 3). Scale bars, 20 μm. Spearman’s rank test, one-way and two-way analysis of variance (ANOVA). Error bars represent SD; *P < 0.05, **P < 0.01, *** *P* < 0.001.

Based on the ChIP-seq results and the GO enrichment analysis from RNA-seq, the potential target PYGL aroused our interest because of its roles in glycogen metabolism and maintenance of redox homeostasis. Further analysis in LNCaP showed that androgen deprivation led to the increase of PYGL mRNA and protein levels (Fig. 3A, B). Furthermore, the increase in PYGL expression was indicative of poor prognosis in patients with CRPC (Fig. S4A, B). Indeed, BRD9 knockdown experiments in combination with androgen deprivation showed that a reduced BRD9 level resulted in an attenuation of PYGL induction (Fig. 3C). Furthermore, BRD9 mRNA expression in clinical PCa cohorts from GSE2443 datasets tended to be positive correlated with PYGL mRNA expression (Fig. 3D). Functionally, PYGL knockdown led to more obvious defects in proliferation and colony formation of CRPC cells relative to androgen-dependent ones (Fig. S4C, D), indicating the requirement of PYGL for progression to incurable PCa. By CHIP-seq analysis, three potential BRD9 binding sites in the promoter of the PYGL gene were predicted (Figs. 3E, S4E). ChIP assay confirmed that BRD9 was recruited to the P3 (nucleotide positions −1960 to −1968) region but not the P1 (nucleotide positions −546 to −554) and P2 (nucleotide positions −877 to −885) region in C4-2B cells (Fig. S4F). The enrichment of BRD9 in the PYGL promoter increased upon androgen deprivation, whereas such increase was mostly ablated by the addition of the ROS scavenger NAC (Fig. 3F), suggesting that the transcriptional regulation of BRD9 towards PYGL was partly dependent on ROS content.

As the PYGL-mediated pathway may serve as a novel mechanism for BRD9 in promoting PCa progression, we next determined whether BRD9 and PYGL are consistent in regulating the malignant phenotype of PCa cells. PYGL overexpression restored the defect of cell proliferation and colony formation caused by BRD9 knockdown (Fig. 3G, H). However, the enhancement of proliferation and colony formation induced by BRD9 overexpression could be attenuated by PYGL knockdown. (Fig. 3I, J). Therefore, PCa cells may exploit the BRD9-PYGL signaling axis to upregulate the antioxidant capacity in favor of tolerating oxidative stress and conferring castration resistance.

### BRD9 transactivates PYGL expression to sustain redox homeostasis

**Fig. 4:**
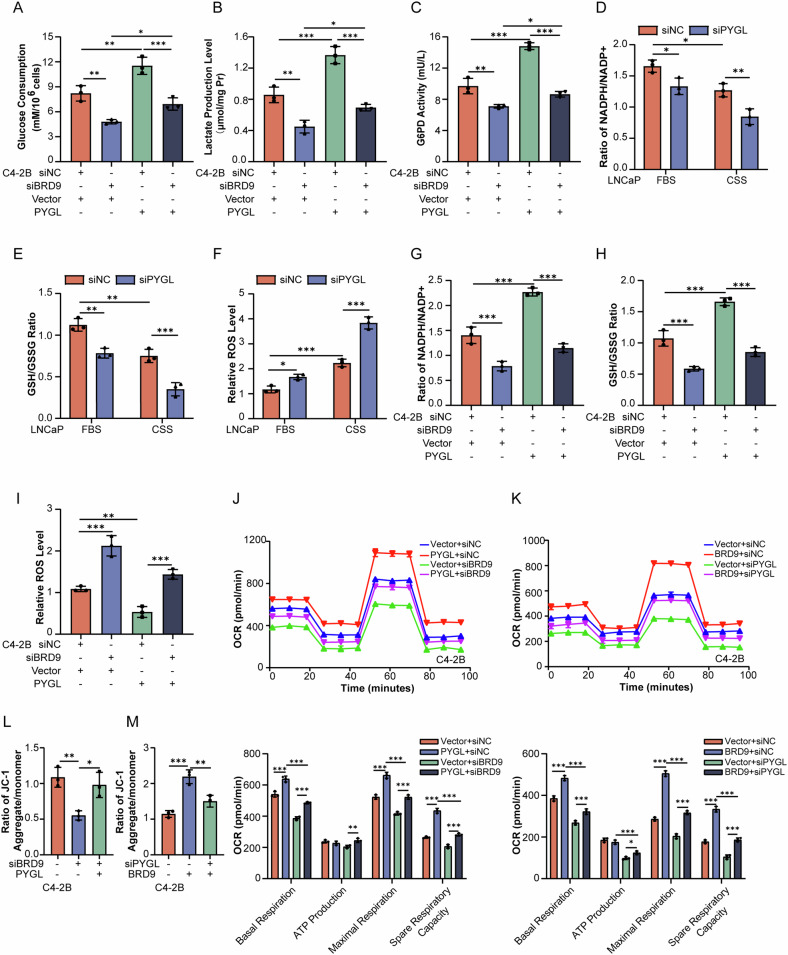
BRD9 maintains REDOX balance via PYGL in PCa cells. **A**–**C** Glucose consumption, lactate production, and G6PD activity in C4-2B cells with transfection of siBRD9 for 24 h and then the PYGL overexpression plasmid for another 24 h (*n* = 3). **D**–**F** NADPH/NADP^+^ ratio, GSH/GSSG ratio, and ROS level in LNCaP cells with PYGL knockdown with or without androgen deprivation (*n* = 3). FBS fatal bovine serum, CSS charcoal-stripped serum. **G**–**I** NADPH/NADP^+^ ratio, GSH/GSSG ratio, and ROS level in C4-2B cells with transfection of siBRD9 for 24 h and then the PYGL overexpression plasmid for another 24 h (*n* = 3). **J**, **K** OCR in C4-2B cells co-transfected with siBRD9 for 24 h and then the PYGL overexpression plasmid for another 24 h or co-transfected with siPYGL siRNA for 24 h and then the BRD9 overexpression plasmid for another 24 h (*n* = 3). Representative recordings of OCR during extracellular flow analysis (“Seahorse”) are shown in the up panel, and quantitative analysis of the calculated basal and maximum respiratory rates, ATP production rate, and spare respiratory capacity are shown in the bottom panel. **L**, **M** Membrane potential in C4-2B cells co-transfected with siBRD9 for 24 h and then the PYGL overexpression plasmid for another 24 h or co-transfected with siPYGL siRNA for 24 h and then the BRD9 overexpression plasmid for another 24 h (*n* = 3). One-way and two-way analysis of variance (ANOVA). Error bars represent SD; *P < 0.05, **P < 0.01, *** *P* < 0.001.

It has been previously observed that PYGL knockdown limited flux into PPP, increased ROS production, markedly induced premature senescence, and impaired tumorigenesis [36]. As expected, the decline of glucose consumption (Fig. 4A), lactate production (Fig. 4B), and G6PD enzyme activity (Fig. 4C) resulting from BRD9 knockdown could be reversed by PYGL overexpression. Moreover, androgen deprivation could induce oxidative stress due to the reduction of NADPH/NADP^+^ ratios (Fig. 4D) and GSH/GSSG ratios (Fig. 4E) and increased ROS production (Fig. 4F) in LNCaP cells, and these inducible effects on oxidative stress became more obvious when PYGL was knockdown (Fig. 4D–F). Furthermore, the inducible effects of BRD9 knockdown on oxidative stress were confirmed by the decreased NADPH/NADP^+^ (Fig. 4G) and GSH/GSSG (Fig. 4H) ratios as well as the increased ROS production (Fig. 4I), whereas these effects were ameliorated by PYGL overexpression (Fig. 4G-I). In contrast, BRD9-induced activation of PPP and the consequent antioxidant effects were only minimally detectable when PYGL was knockdown (Fig. S5A–F). The activity of mitochondria was also analyzed, and importantly, overexpression of PYGL could restore the decline of OCR and mitochondrial membrane potential caused by BRD9 knockdown in C4-2B cells (Fig. 4J, L), whereas the protective effects of BRD9 overexpression on the above parameters tended to be less pronounced when PYGL was knockdown (Fig. 4K, M). These data further support that BRD9 is responsive to oxidative stress and behaves as an antioxidant factor to maintain the redox balance in PCa.

Further functional experiments demonstrated a high level of BRD9 to be advantageous for cells under oxidative stress. We treated control cells and cells with BRD9 overexpression with hydrogen peroxide (H_2_O_2_) and measured cell survival. Under H_2_O_2_ exposure, PCa cells with elevated BRD9 and PYGL expression displayed increased survival when compared with parental control cells (Fig. S6A). The promotive effect of BRD9 overexpression on the growth of PCa cells was significantly attenuated by silencing PYGL (Fig. S6A). In contrast, overexpression of PYGL could reverse the decline of cell proliferation caused by BRD9 knockdown (Fig. S6B). CP-320626, a specific inhibitor of PYGL, was also introduced into the culture medium of C4-2B cells, and the response to CP-320626 recapitulated our results obtained with the siRNA-mediated PYGL knockdown, even in the presence of BRD9 overexpression (Fig. S7A, B). When castrated mice bearing C4-2B with BRD9 overexpression or not were treated with CP-320626, the BRD9-induced increase of tumor growth was significantly inhibited, as shown by tumor growth, tumor volume, and the proliferation index Ki67 (Fig. S7C–F). These data provide compelling evidence that BRD9 protects PCa against the cytotoxic effects of oxidative stress and that BRD9 is required for the advantageous upregulation of PYGL under androgen-deprived conditions, thereby helping to maintain an antioxidant state in the PCa cells; these findings may explain the superior capacity of CRPC-like PCa cells to survive under oxidative stress.

### BRD9 is responsive to oxidative stress for S-glutathionylation

**Fig. 5:**
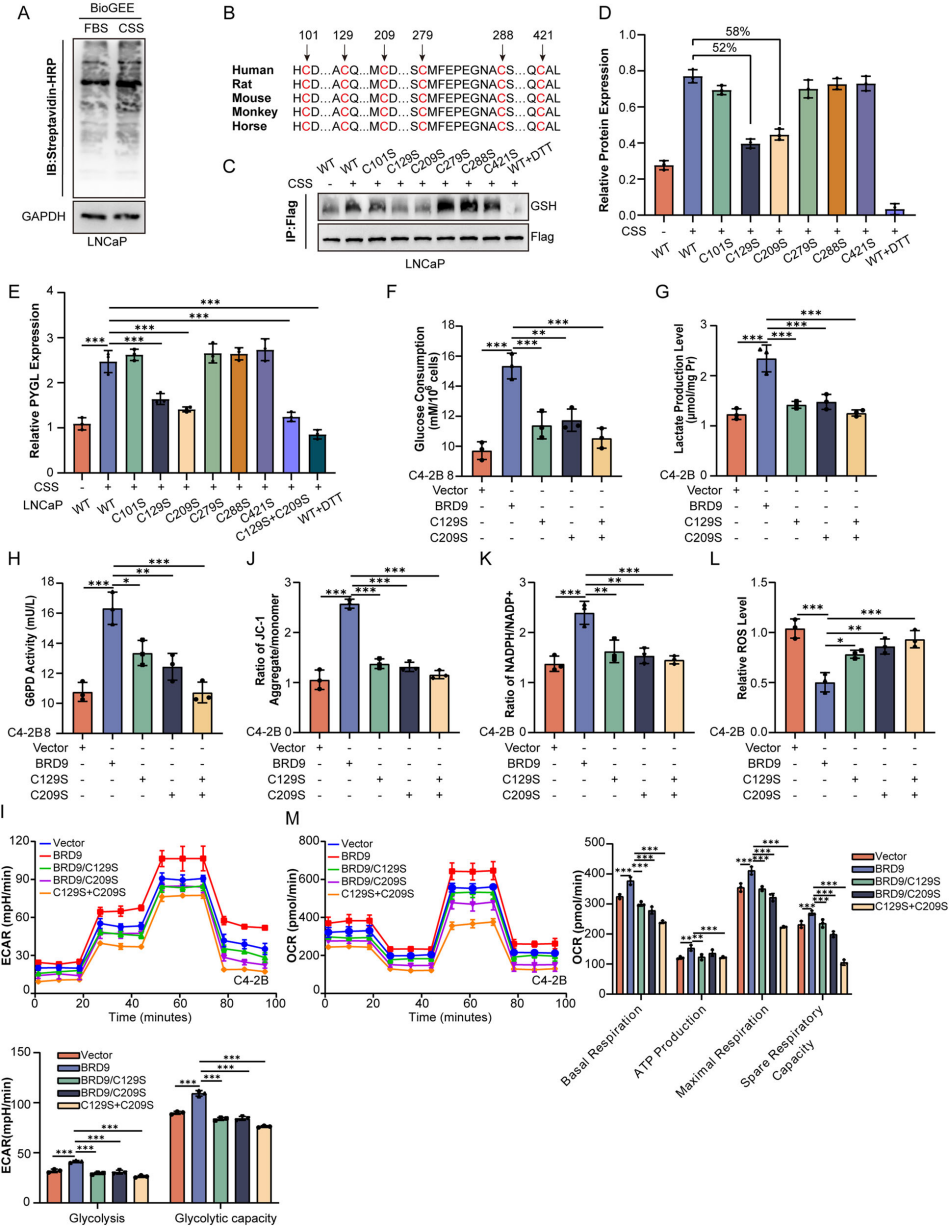
BRD9 is S-glutathionylated in response to oxidative stress. **A** LNCaP cells with or without androgen deprivation, loaded into BioGEE (250 μM) for 4 h, and cell lysis products were analyzed by nonreducing SDS-PAGE followed by western blotting analysis with horseradish peroxidase-labeled streptavidin (*n* = 3). BioGEE, Glutathione Ethyl Ester, Biotin Amide. **B** Alignment of BRD9 amino acid sequences from different mammalian species. Conserved cysteine residues are indicated by red font. **C** Immunoprecipitation assay to detect S-glutathionylation of BRD9. LNCaP cells were transfected with a Flag-BRD9 vector containing the wild-type BRD9 sequence (WT), or vectors expressing Flag-BRD9 with mutations of C101 (C101S), C129 (C129S), C209 (C209S), C279 (C279S), C288 (C288S) or C421 (C421S). Immunoprecipitation was performed with anti-Flag antibody, and glutathionylation was detected by anti-GSH antibody with or without androgen deprivation (*n* = 3). DTT (dithiothreitol) was used as a reducing reagent. **D** A quantitative analysis of the levels of glutathionylation calculated capacity. **E** Relative PYGL mRNA expression in LNCaP cells expressing BRD9 mutants. LNCaP cells expressing wild-type Flag-BRD9 vector (WT) or Flag-BRD9 with mutations (as indicated for (**C**)) were incubated with or without androgen deprivation (*n* = 3). DTT dithiothreitol. **F**–**H** Glucose consumption, lactate production, and G6PD activity in C4-2B cells overexpressing BRD9 or mutated BRD9. **I** Measurement of ECAR in indicated C4-2B cells overexpressing BRD9 or mutated BRD9 (*n* = 3). Representative recordings of ECAR during extracellular flow analysis (“Seahorse”) are shown in the up panel, and quantitative analysis of the calculated Glycolysis and Glycolytic capacity are shown in the bottom panel. **J**–**L** Membrane potential, NADPH/NADP^+^ ratio, and ROS level in C4-2B cells overexpressing BRD9 or mutated BRD9 (*n* = 3). **M** Measurement of OCR in indicated C4-2B cells overexpressing BRD9 or mutated BRD9 (*n* = 3). Representative recordings of OCR during extracellular flow analysis (“Seahorse”) are shown in the left panel, and quantitative analysis of the calculated basal and maximum respiratory rates, ATP production rate, and spare respiratory capacity are shown in the right panel. One-way analysis of variance (ANOVA). Error bars represent SD; *P < 0.05, **P < 0.01, *** *P* < 0.001.

As our collective results pointed to oxidative stress as a key factor in inducing BRD9 to promote PCa progression and the effects of ROS signaling are achieved through oxidative modification of various ROS substrates, we decided to analyze the effect of ROS on BRD9. With the help of glutathionylation assay, reversible thiol oxidation (glutathionization) was found to be increased when LNCaP cells were challenged by androgen deprivation (Fig. 5A).

To determine the specific amino residues that were oxidized, we performed sequence alignment of BRD9 homologs from different species and identified six cysteine residues that are highly conserved across species, cysteine 101, cysteine 129, cysteine 209, cysteine 279, cysteine 288 and cysteine 421 (Fig. 5B). The cysteine residue of BRD9 was mutated to serine for cell biology and biochemical experiments. The levels of glutathionization of mutant BRD9^C129S^ and BRD9^C209S^ declined by more than 50% (Fig. 5C,D), indicating that Cys129 and Cys209 are the major residues involved in the glutathionization of BRD9. Moreover, the double mutant BRD9^C129S/C209S^ barely induced PYGL expression (Fig. 5E), and consequently failed to increase glucose consumption (Fig. 5F), lactate production (Fig. 5G), G6PD enzyme activity (Fig. 5H) and extracellular acidification rate (ECAR) (Fig. 5I) when compared to the wild-type or single-mutation protein. Furthermore, the inability of double mutant BRD9^C129S/C209S^ hardly increase mitochondrial membrane potentials (Fig. 5J), NADPH/NADP^+^ ratio (Fig. 5K), OCR (Fig. 5M) and couldn’t reduce ROS level (Fig. 5L). Collectively, these results suggest that BRD9 responds to oxidative stress by serving as a substrate for S-glutathionylation, which leads to enhanced redox-protective changes from the onset of androgen deprivation.

### BRD9 requires NFYA to affect redox balance

**Fig. 6:**
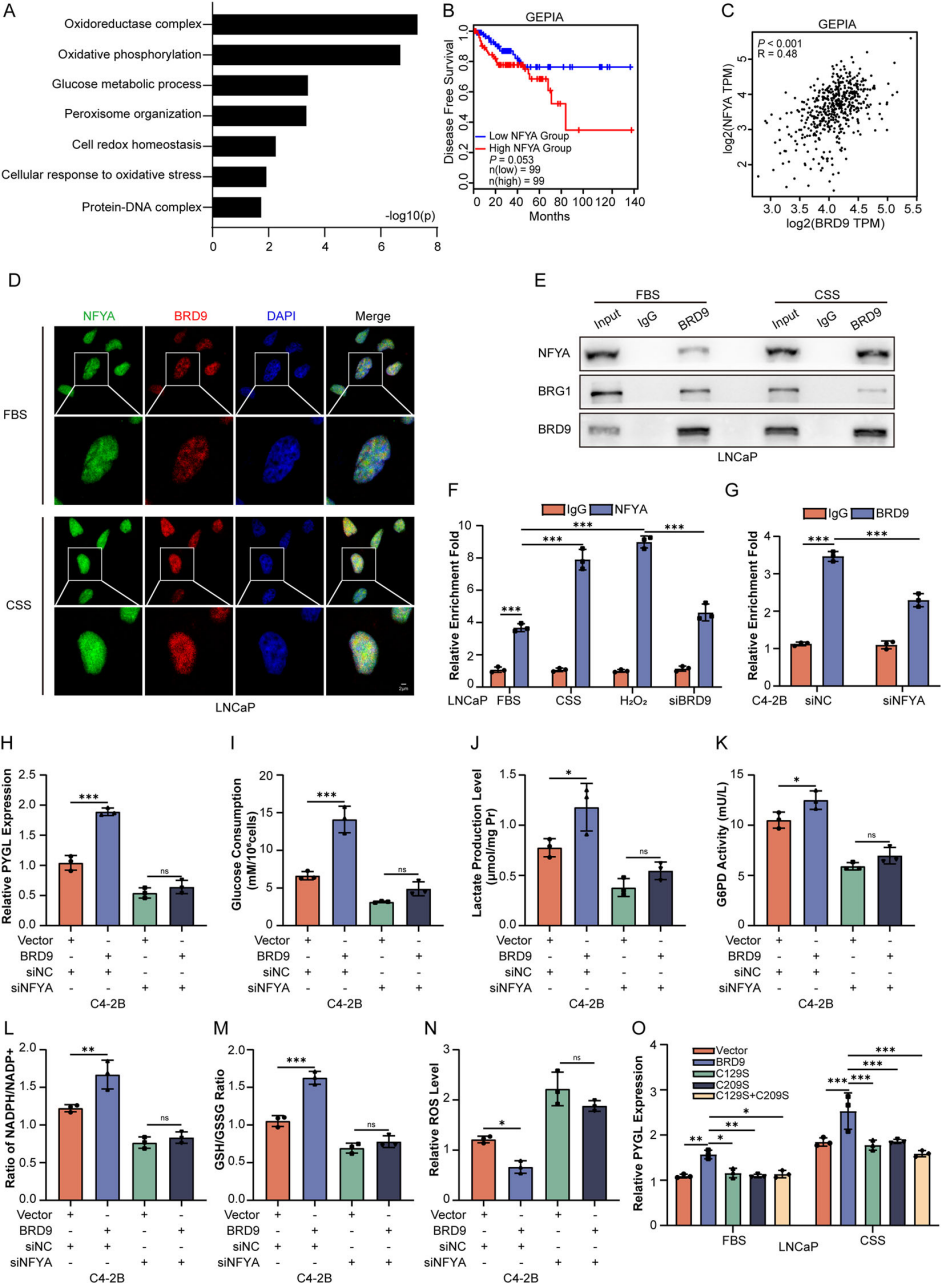
BRD9 maintains REDOX balance and PPP activity in PCa cells through NFYA. **A** KEGG pathway gene set enrichment analysis of the potential BRD9 interactors identified by co-immunoprecipitation assays and mass spectrometry. **B** Kaplan-Meier survival analysis of PCa cases from the GEPIA prostate cohort according to relative expression of NFYA. **C** Correlation between the relative levels of BRD9 and NFYA mRNA transcripts in prostate cancer tissues of the GEPIA database. **D** Localization of BRD9 (red) and NFYA (green) in LNCaP cell nuclei was verified by immunofluorescent staining with or without androgen deprivation. Magnified images from the regions marked by rectangles in the top panel were showed in the bottom panel (*n* = 3). Scale bars, 2 μm. **E** Binding potential between BRD9 and NFYA or BRG1 in the whole cell lysates was determined by co-immunoprecipitation assays in LNCaP cells with or without androgen deprivation (*n* = 3). IgG served as negative control. ChIP analysis using LNCaP or C4-2B cells to validate (**F**) NFYA or (**G**) BRD9 enrichment on the PYGL promoter (*n* = 3 for each panel). **H** Relative levels of PYGL mRNA expression in C4-2B cells transfected with empty vector or the BRD9 overexpression plasmid with or without NFYA suppression (*n* = 3). **I**–**N** Glucose consumption, lactate production, G6PD activity, NADPH/NADP^+^ ratio, GSH/GSSG ratio, and ROS level in C4-2B cells transfected with empty vector or BRD9 overexpression plasmid with or without NFYA suppression (*n* = 3). **O** Relative levels of PYGL mRNA expression in LNCaP cells transfected with wild-type Flag-BRD9 vector (WT), or vectors expressing Flag-BRD9 with single or double cysteine mutations with or without androgen deprivation (*n* = 3). FBS fatal bovine serum, CSS charcoal-stripped serum. Kaplan–Meier survival analysis, Spearman’s rank test, one-way and two-way analysis of variance (ANOVA). Error bars represent SD; *P < 0.05, **P < 0.01, *** *P* < 0.001.

To elucidate the antioxidant functions of BRD9, a co-immunoprecipitation experiment, followed by a proteomic analysis of individual spots, was performed. We identified 485 proteins that potentially interact with BRD9, including the components of the SWI/SNF complex, SMARCE1 and SMARCB1. By bioinformatics analysis, the BRD9 proteome identified here was predicted to affect protein networks of glucose metabolism and endogenous antioxidant mechanisms (Fig. 6A). Among the candidate interactor, NFYA has arouse our attention as it’s demonstrated to promote PCa progression via regulating PCa cell metabolism, notably respiration and aerobic glycolysis [37]. Interestingly, NFYA might be important for compensatory pathways maintaining normal cellular oxidative stress levels [38].

PCa patients with low expression of NFYA tended to have had longer DFS compared with those with high expression of NFYA using the GEPIA database, although there was no statistically significant difference (*P* = 0.053, Fig. 6B). Additionally, NFYA mRNA expression in PCa samples tend to be positively correlated with BRD9 mRNA expression in GEPIA datasets (Fig. 6C). Immunofluorescent staining showed co-localization of BRD9 and NFYA in the nucleus from PCa cells (Fig. 6D). Moreover, androgen deprivation enhanced the interaction between BRD9 and NFYA, whereas the binding of BRD9 to its classical partner, BRG1, was reduced (Fig. 6E). Consistently, the recruiting of NFYA towards the PYGL promoter increased when PCa cells were challenged by androgen deprivation and H_2_O_2_, whereas this increase could be attenuated by BRD9 knockdown (Fig. 6F). Indeed, silencing NFYA could also disrupt the binding of BRD9 to the PYGL promoter (Fig. 6G). Detailed analysis showed that ectopic expression of BRD9 could only minimally induce PYGL expression (Fig. 6H), activate PPP metabolism (Fig. 6I–K), or initiate an antioxidant effect (Fig. 6L–N) when NFYA was absent. These results support a role for NFYA in BRD9-mediated PYGL induction and antioxidant activity.

We continued to analyze the consequences of androgen deprivation-mediated BRD9 oxidation on NFYA and proposed that the androgen deprivation-derived ROS oxidation of BRD9 may facilitate the transcriptional effects of NFYA. As expected, wild-type BRD9 interacted with NFYA more intensely upon exposure to androgen deprivation. In contrast, BRD9^C129S/C209S^ failed to interact with NFYA (Fig. S8), induce PYGL expression (Fig. 6O), activate glycolysis-PPP metabolism or clear ROS in PCa cells (Fig. 5F–L). These findings further support our hypothesis that oxidized BRD9 can relay the redox stress signals from a harsh cellular environment to induce metabolic preference towards the glycogen degradation system in PCa.

### Targeting BRD9 is effective in delaying PCa progression

**Fig. 7:**
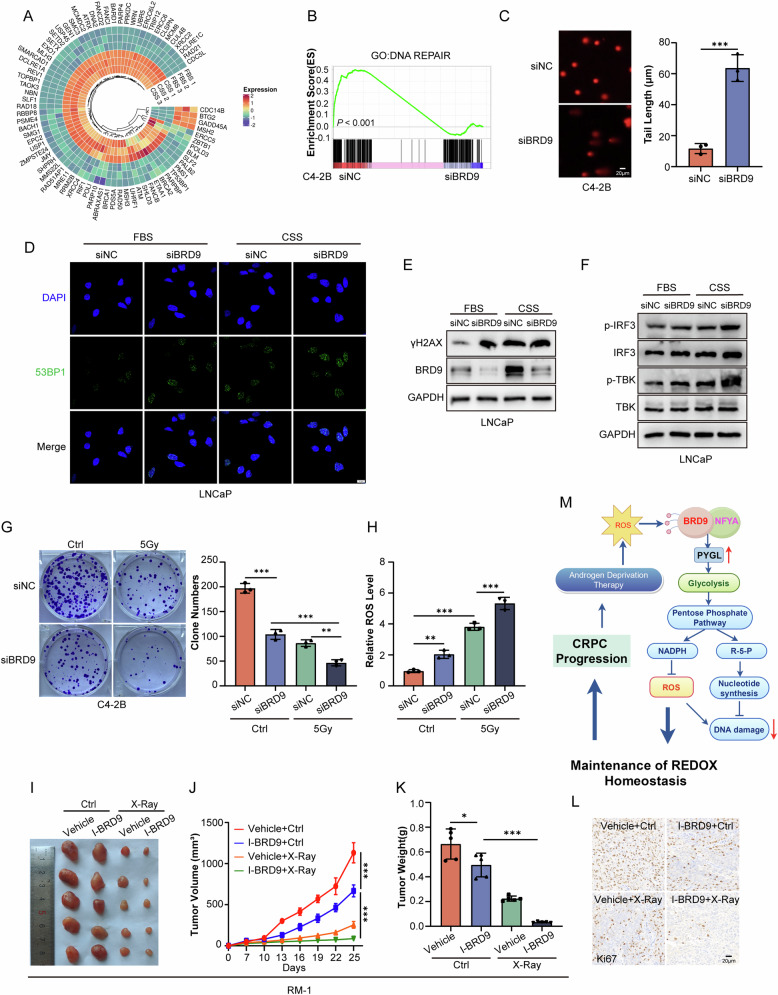
Targeting BRD9 is effective in delaying CRPC progression. **A** Changes in expression in DNA repair-related genes LNCaP cells before and after androgen deprivation. **B** Gene Set Enrichment Analysis (GSEA) enrichment plot of the DNA repair-related gene sets in C4-2B cells with or without BRD9 silencing (siBRD9 or siNC, respectively). **C** Comet assay in C4-2B cells with or without BRD9 silencing (siBRD9 or siNC, respectively) (*n* = 3). A representative image is shown in the left panel, and quantitative analysis of the tail length is shown in the right panel. Scale bars, 20 μm. **D** Immunofluorescence staining assay of DNA double-stranded breaks (DSBs) in LNCaP cells with BRD9 knockdown with or without androgen deprivation (*n* = 3). Scale bars, 10 μm. **E** DSBs-associated protein levels and **F** phosphorylation levels of IRF3 and TBK proteins in LNCaP cells with BRD9 knockdown with or without androgen deprivation. (*n* = 3). **G** Proliferation was measured in C4-2B cells with or without BRD9 silencing (siBRD9 or siNC, respectively) in response to X-ray irradiation (*n* = 3). Representative images are shown in the left panel, and quantitative analysis is shown in the right panel. **H** ROS level in C4-2B cells with or without BRD9 silencing (siBRD9 or siNC, respectively) in response to X-ray irradiation (*n* = 3). Gy, Gray. **I**–**L** C57BL/6 mice bearing xenografts (RM-1 cells) following treatment with I-BRD9 or radiation therapy (*n* = 5/group). Tumor volume was measured (**J**) and then weighed (**K**) after mice were euthanized. Immunohistochemistry staining of Ki67 (**L**) on tumor slides from each group is shown. **M** A putative schematic diagram illustrating the role of BRD9 in contributing to CRPC. One-way and two-way analysis of variance (ANOVA). Error bars represent SD; *P < 0.05, **P < 0.01, *** *P* < 0.001.

Excessive ROS production can induce DNA damage and cell apoptosis [39]. Therefore, we compared gene expression before and after androgen deprivation in PCa cells and found that the expression of genes related to DNA repair was significantly increased in the androgen-deprived group (Fig. 7A). Importantly, GSEA functional annotation confirmed that the DEGs in response to BRD9 knockdown were enriched for DNA repair processes (Fig. 7B). We then assessed whether reduction of endogenous BRD9 influences DNA damage. BRD9 knockdown led to severe DNA damage in C4-2B cells, as evidenced by the increase of the tail length in the comet assay (Fig. 7C). BRD9 silencing induced DNA damage with significant activation of double-strand breaks (DSBs) markers 53BP1 (Fig. 7D) and γH2AX (Fig. S9A), and similar detrimental effects became more obvious under androgen deprivation in LNCaP cells. A similar tendency was also observed in detecting the DSBs markers γH2AX by Western blotting (Fig. 7E).

Inactivation of BRD9 was sufficient to induce ROS production, suppress DNA repair factors, and consequently lead to the accumulation of nuclear DNA (Fig. S9B) and mitochondrial DNA in the cytosol (Fig. S9C), we proposed that the accurate targeting of BRD9 in tumor cells may be a strong potential therapeutic strategy to improve the efficacy of radiotherapy. Previous studies demonstrated that cyclic GMP-AMP synthase (cGAS) bound to dsDNA and then activated the cGAS-STING pathway, the latter of which is associated with the therapeutic effects of radiotherapy [40]. As expected, BRD9 knockdown activated cGAS-STING signaling as the phosphorylated levels of its components (TBK1 and IRF3) increased, especially under androgen-deprived conditions (Fig. 7F). The production of IFNβ, CCL5, STING, and CXCL10 was also significantly increased when BRD9 was silenced with or without radiation treatment (Fig. S10A–D). However, this effect could be reversed by PYGL overexpression (Fig. S10E–H). Similarly, BRD9 knockdown combined with radiotherapy could synergistically induce DNA damage, as evidenced by the increased expression of γH2AX and 53BP1, whereas ectopic PYGL expression efficiently attenuated this induction of DNA damage (Fig. S11). Therefore, the antioxidant role of BRD9 empowers PCa cells resistance to DNA damage that activates the cGAS-STING signaling pathway.

In further support of the relevance of these findings for therapeutic purposes, BRD9 knockdown combined with radiotherapy significantly inhibited cell proliferation and increased intracellular ROS production in C4-2B cells (Fig. 7G, H). Additionally, established PCa derived from mouse RM-1 cells injection were treated with local radiation and I-BRD9, and after irradiation, tumors lacking BRD9 were notably restricted in their growth compared to the control group (Fig. 7I–L). Overall, our findings indicate that tumor cell-intrinsic BRD9 orchestrates metabolic reprogramming and that BRD9 ablation may be a viable avenue for improving the effectiveness of radiotherapy (Fig. 7M).

## Discussion

PCa cells reprogram their metabolism to gain survival advantages when confronted by androgen deprivation or oxidative stress [41]. This study identified BRD9 as a metabolic modulator that directs glucose utilization toward an antioxidant pathway. Importantly, BRD9 inhibition limited the input of glucose into the PPP, confined antioxidant activity, disrupted mitochondrial respiration, and strongly impaired PCa progression, thus serving as a promising target for clinical treatment.

Androgen deprivation encourages metabolic reprogramming of PCa cells to survive and proliferate [42]. Glycogen degradation by PYGL serves as an important pathway for metabolic reprogramming in cancer cells because it modulates the optimal function of PPP and thereby cellular fate [36]. In this study, we demonstrated that, in response to androgen deprivation, BRD9 could induce PYGL expression, activate glycolysis and PPP, and increase NADPH production, which enabled more efficient clearance of ROS and ultimately sustained survival of PCa cells. This finding is also consistent with the NADPH-dependent antioxidant activity of PPP in tumor growth and drug resistance [43, 44]. The metabolic consequence of disrupting glycogen breakdown by targeting BRD9 was to hinder channeling glycogen-derived glucose to PPP and therefore could be referred to as the “glycogen shunt”. Taken together, the data presented here identify glycogen degradation not only as a hallmark of PCa metabolism but also as a potent, disease-driving oncogenic pathway activated by BRD9 to benefit PCa survival and finally lead to an androgen-deprivation or castration-resistant phenotype.

Recently, mitochondrial oxidative stress was found to activate PPP, further supporting the intimate relation between them [45]. Furthermore, the activation of PPP metabolites and the consequent protection of OXPHOS along with an enhancement of free radical elimination highlight the coordinated and profound roles of BRD9 in cellular adaptation to androgen deprivation. Notably, in androgen-deprived PCa cells, oxidative stress-responsive BRD9 seems to predominantly exploit glycogen utilization to allow a metabolic switch toward PPP with an antioxidant state. Therefore, BRD9 behaves as an important transducer to relay oxidative stress signals from mitochondria to PPP. Furthermore, the pattern of BRD9 interaction is complex and depends on an active metabolic pathway influenced by androgen availability and the redox state. Such dynamic changes in BRD9’s regulatory roles and its target specificity support the functioning of BRD9 as a metabolic checkpoint to fine-tune the energetic state and progressive fate of PCa cells.

Metabolic aberrations in cancer cells leading to ROS accumulation can initiate carcinogenesis and the malignant transformation of cells [46–48]. However, excessive ROS production induces oxidative damage to mitochondrial and nuclear DNA integrity and even cell viability [49]. Therefore, a strategy that generates excess ROS could be translated into a therapeutic approach to induce DNA damage and cancer cell death [50, 51]. The major therapeutic feature of radiation is the induction of toxic oxidative damage resulting from excessive production of ROS [52, 53]. Additionally, the release of mitochondrial DNA by mitochondrial outer membrane permeabilization promotes the secretion of type I interferon by malignant cells [54], which is required for the activation of optimal anticancer immune responses upon radiation therapy and chemotherapy [55, 56]. In particular, patients with advanced PCa showed overwhelming resistance to immune checkpoint inhibitors, such as the one targeting PD-1 and its ligand PD-L1 [57, 58]. PYGL is responsible for breaking down glycogen into the glycolytic pathway [59], or the PPP, which is of great significance in the radioprotection of tumor cells [39, 60, 61]. Previous studies have demonstrated that the acute knockdown of PYGL and the subsequent glycogen accumulation in malignant gliomas constricted the flux into PPP which provoked excessive ROS accumulation. Ultimately, this led to premature senescence and impaired tumorigenesis [36]. As expected, the increase of ROS accumulation because of BRD9 inhibition could enhance the radiosensitivity of PCa cells. As energy or nucleotide biosynthesis is important for the repair of DNA damage [62, 63], it’s conceivable that, besides NADPH, they also account for the response of BRD9 to radiation therapy in PCa.

Together, these findings reveal a striking role for BRD9 as a prominent metabolic checkpoint that intricately regulates glycogen utilization to improve metabolic plasticity in favor of mitochondrial respiration that endows a CRPC phenotype. Importantly, BRD9 is an actionable target via pharmacologic or genetic strategies, pointing to tractable means to improve a broad spectrum of castration or radiation therapies.

## Supplementary information

Supplementary materials

## Data Availability

All data and materials during the current study are available from the corresponding author upon reasonable request.
