# Supplementary material for: Prostate cancer exploits BRD9-driven metabolic reprogramming to shape the aggressive phenotype
Source: Cell Death Dis. 2025 Apr 22;16(1):326. doi: 10.1038/s41419-025-07561-9 (PMC12015546; doi:10.1038/s41419-025-07561-9)

Supplementary Figures and Legends

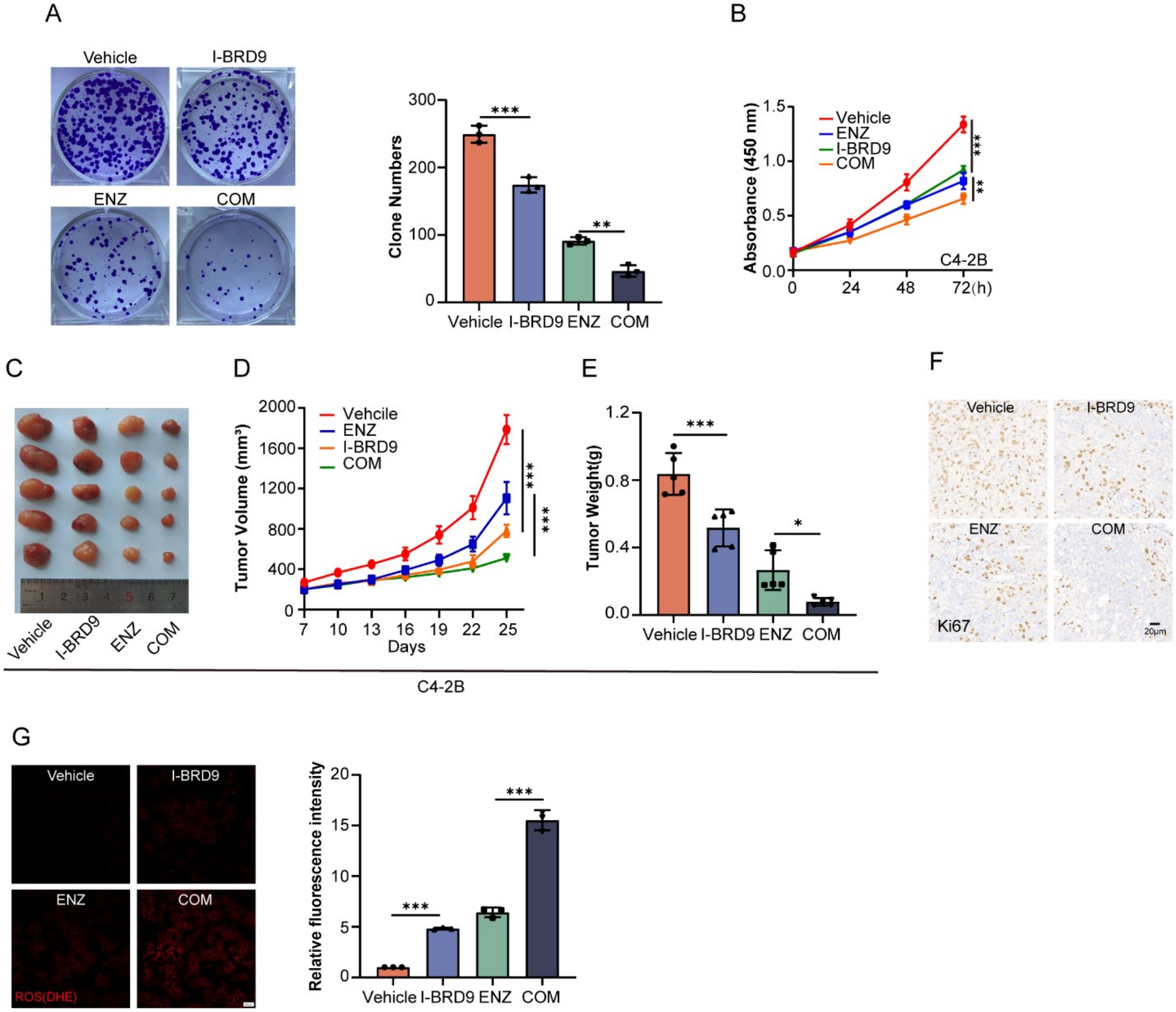

Fig. S1 I-BRD9 inhibits CRPC progression in vitro and in vivo.

A, B Colony formation and CCK-8 assays in C4-2B cells treated with enzalutamide (10 μM), I-BRD9 (10 μM), or a combination of enzalutamide and I-BRD9 (n = 3/group). h, hours. ENZ, enzalutamide. COM, combination. **C, D, E, F** Castrated mice bearing xenografts (C4-2B cells) were treated with enzalutamide (10 mg/kg), I-BRD9 (20 mg/kg), or a combination of enzalutamide and I-BRD9 (n = 5/group). The volume (C, D) and weight (E) of the tumors were measured after euthanizing the mice. (F) IHC staining of Ki67 on tumor sections treated with enzalutamide, I-

BRD9, or a combination of enzalutamide and I-BRD9 (n = 5/group) Scale bars, 20 μm. **G** DHE staining was used to determine ROS generation in tumor sections. Representative images are shown in the left panel, and quantitative analysis is shown in the right panel (n = 3). Scale bars, 50 μm. ENZ, enzalutamide; COM, combination. One-way and two-way analysis of variance (ANOVA). Error bars represent SD; *$P < 0.05$, **$P < 0.01$, *** $P < 0.001$.

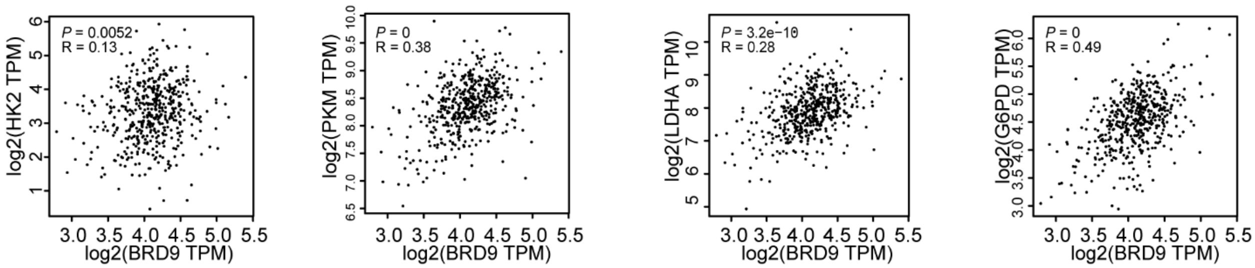

Fig. S2 BRD9 is associated with glycolytic molecules in PCa cells.

Correlation between the relative expression levels of HK2, PKM, LDHA, or G6PD and BRD9 mRNA transcripts in PCa tissues from the GEPIA database. Spearman's rank test was used to analyze the relationship between gene expression levels.

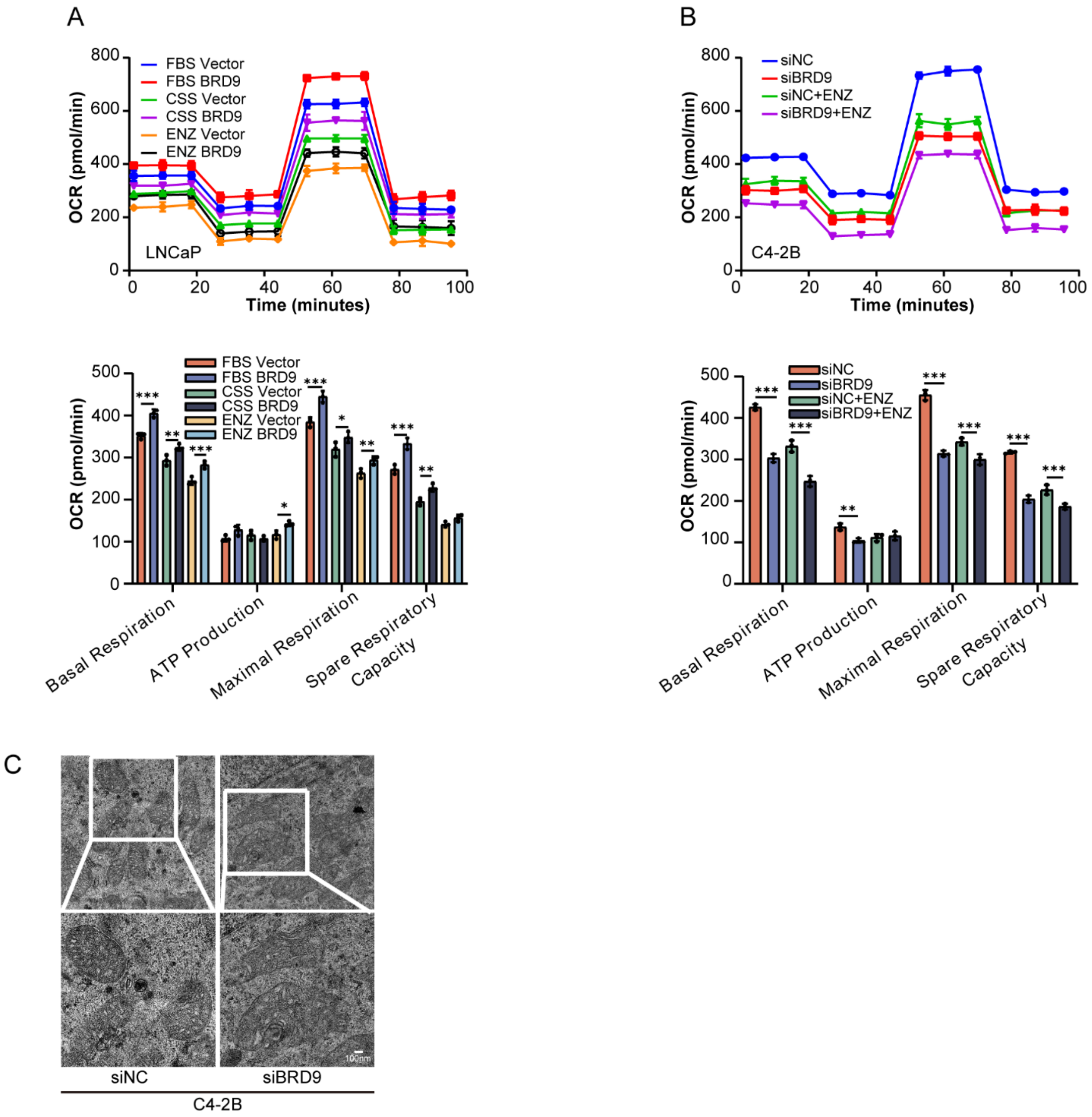

Fig. S3 BRD9 affects mitochondrial function and morphology.

A, B Measurement of OCR in PCa cells with BRD9 (A) overexpression or (B) knockdown in response to androgen deprivation and enzalutamide (10 μM) (n = 3). Representative recording of OCR during extracellular flow analysis (“Seahorse”) is shown in the up panels, and quantitative analysis of the calculated basal and maximum respiratory rates, ATP production rate, and spare respiratory capacity are shown in the bottom panels. **C** Electron microscopy images of

mitochondrial morphology in C4-2B cells with BRD9 knockdown. Magnified images from the regions marked by rectangles in the top panel was showed in the bottom panel. Scale bars, 100 nm. FBS, fetal bovine serum. CSS, charcoal-stripped serum. ENZ, enzalutamide. Two-way analysis of variance (ANOVA). Error bars represent SD; **P* < 0.05, ***P* < 0.01, *** *P* < 0.001.

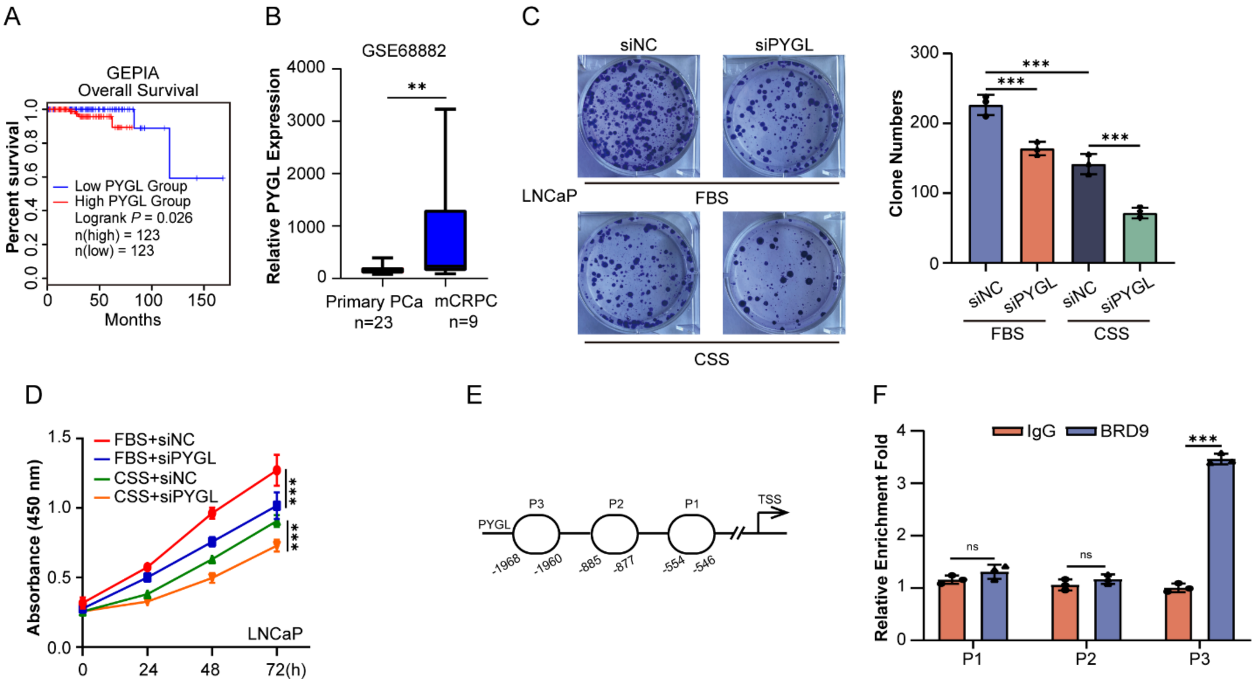

Fig.S4 PYGL serves as a direct target for BRD9 and is associated with PCa progression.

A Kaplan-Meier survival analysis of PCa cases from the GEPIA cohort according to high and low PYGL expression in PCa tissues. **B** Comparison of the expression levels of PYGL in various prostate tissues (GSE68882 dataset). **C-D** Colony formation and CCK-8 assays in LNCaP cells with PYGL knockdown with or without androgen deprivation. Representative images are shown in the left panel, and quantitative analysis is shown in the right panel (n = 3). FBS, fatal bovine serum. CSS, charcoal-stripped serum. **E** Three putative BRD9 binding sites in the PYGL promoter based on ChIP-seq data. TSS, transcriptional start site. **F** ChIP analysis of BRD9 enrichment in the PYGL promoter in C4-2B cells (n = 3). Purified rabbit IgG was used as the negative control. Kaplan-Meier survival analysis, two-tailed unpaired t-test, one-way and two-way analysis of

variance (ANOVA). Error bars represent SD; *$P < 0.05$, **$P < 0.01$, *** $P < 0.001$.

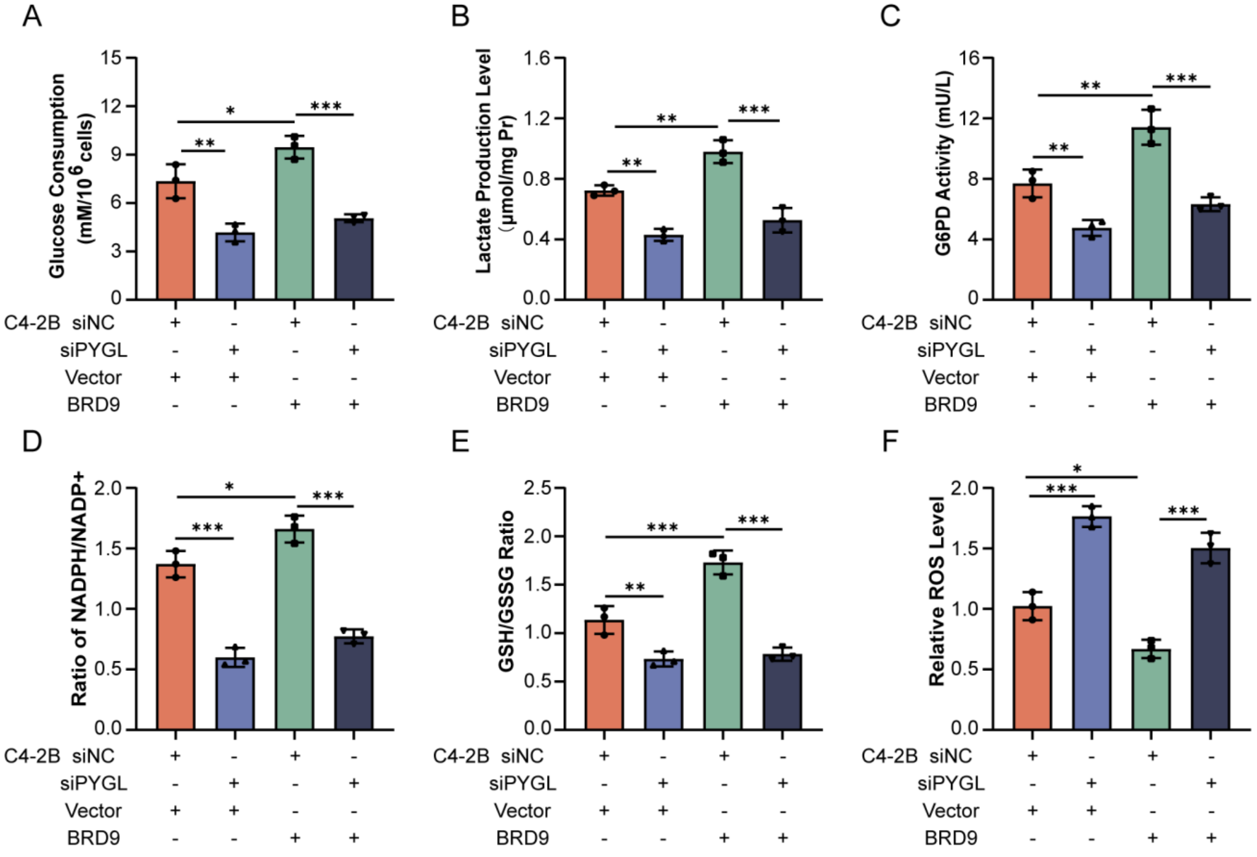

Fig.S5 BRD9 maintains REDOX balance via PYGL in PCa cells.

A-C Glucose consumption, lactate production, and G6PD activity in C4-2B cells transfected with siPYGL or siNC for 24 hours and subsequent transfection with the BRD9 overexpression plasmid for another 24 hours (n = 3). **D-F** NADPH/NADP$^+$ ratio, GSH/GSSG ratio, and ROS level in C4-2B cells transfected with siPYGL or siNC for 24 hours and subsequent transfection with the BRD9 overexpression plasmid for another 24 hours (n = 3). One-way analysis of variance (ANOVA). Error bars represent SD; *$P < 0.05$, **$P < 0.01$, *** $P < 0.001$.

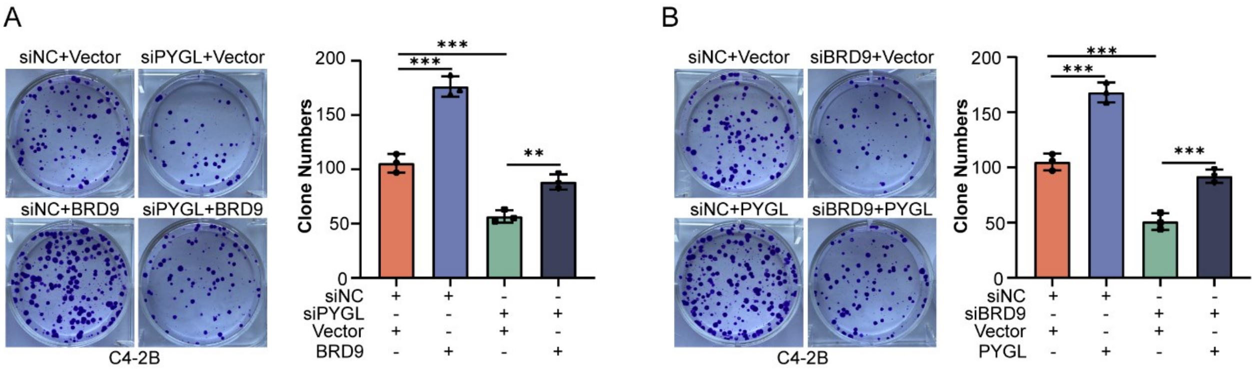

Fig.S6 BRD9 has a protective effect on cells under oxidative stress.

A-B Under H_2O_2 stimulation, colony formation was assessed in C4-2B cells transfected with siPYGL or siNC for 24 hours and then the BRD9 overexpression plasmid for another 24 hours (n = 3) or (B) transfected with siBRD9 or siNC for 24 hours and then the PYGL overexpression plasmid for another 24 hours (n = 3). A representative image is shown in the left panel, and quantitative analysis of the tail length is shown in the right panel. One-way analysis of variance (ANOVA). Error bars represent SD; $^{*}P < 0.05$, $^{**}P < 0.01$, $^{***}P < 0.001$.

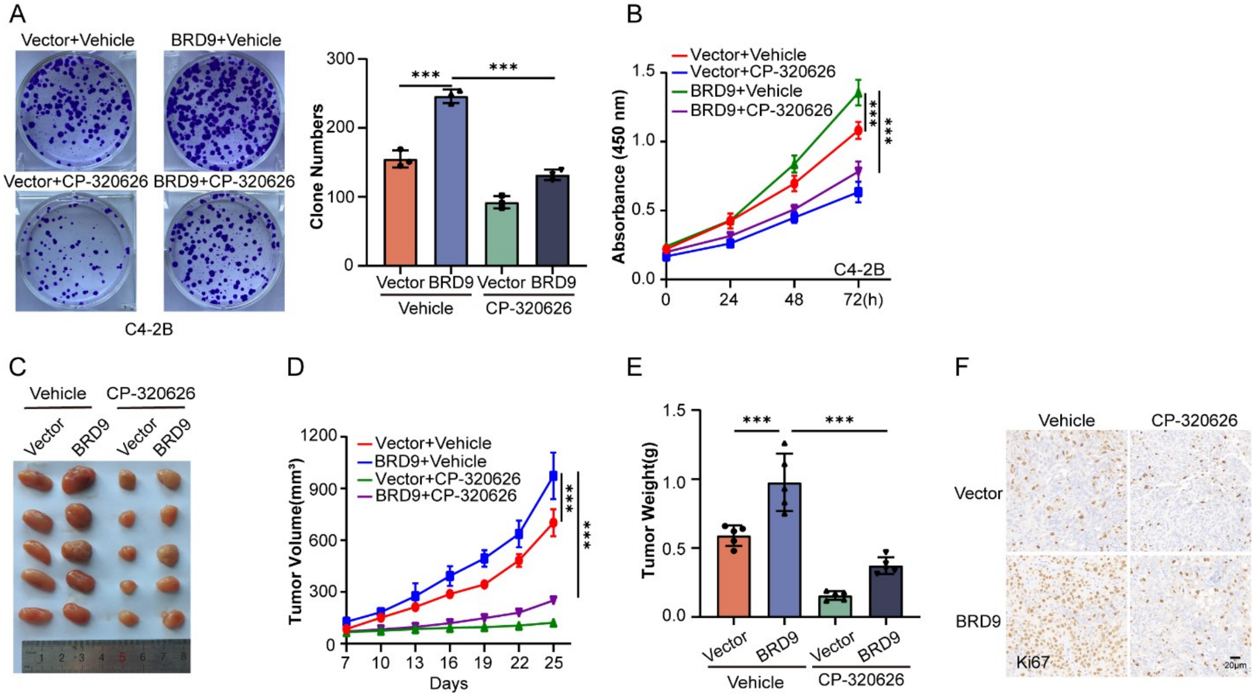

Fig.S7 CP-320626 inhibits CRPC progression in vitro and in vivo.

A-B CCK-8 and colony formation assays of C4-2B cells with BRD9 overexpression in response to CP-320626 (25 μM) exposure (n = 3). A representative image is shown in the left panel, and quantitative analysis of the tail length is shown in the right panel. h, hours. **C-F** Castrated mice with xenografts (C42B-Vector or C42B-BRD9) received CP-320626 treatment (100 mg/kg) or vehicle. Tumor volume was measured every three days. Tumors were collected after euthanizing the mice, and tumor volume (C, D) and weight (E) were measured. IHC staining of Ki67 (F) on tumor sections from each group is shown. Scale bars, 20 μm. One-way and two-way analysis of variance (ANOVA). Error bars represent SD; $*P < 0.05$, $**P < 0.01$, $*** P < 0.001$.

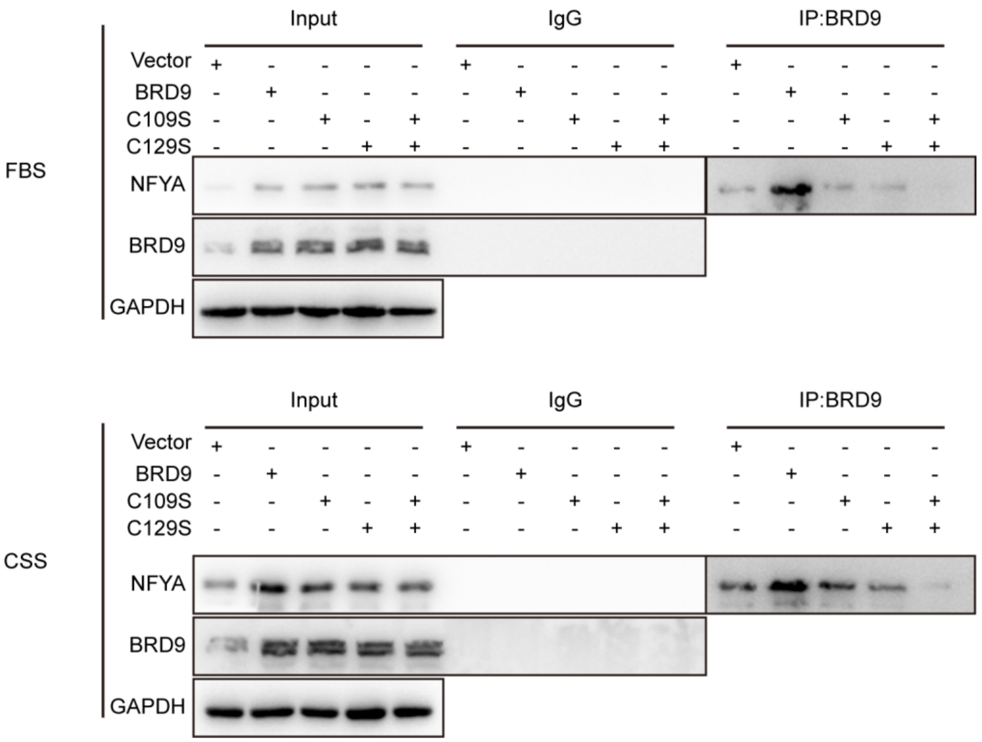

Fig.S8 Co-immunoprecipitation analysis of the binding of BRD9 to NFYA.

The binding potential between BRD9 and NFYA was determined by Co-IP assays in LNCaP cells in response to androgen deprivation with empty vector or plasmids expressing wild-type BRD9 or BRD9 mutants (C109S and C129S). IgG was used as a negative control. GAPDH was used as a loading control. FBS, fatal bovine serum. CSS, charcoal-stripped serum.

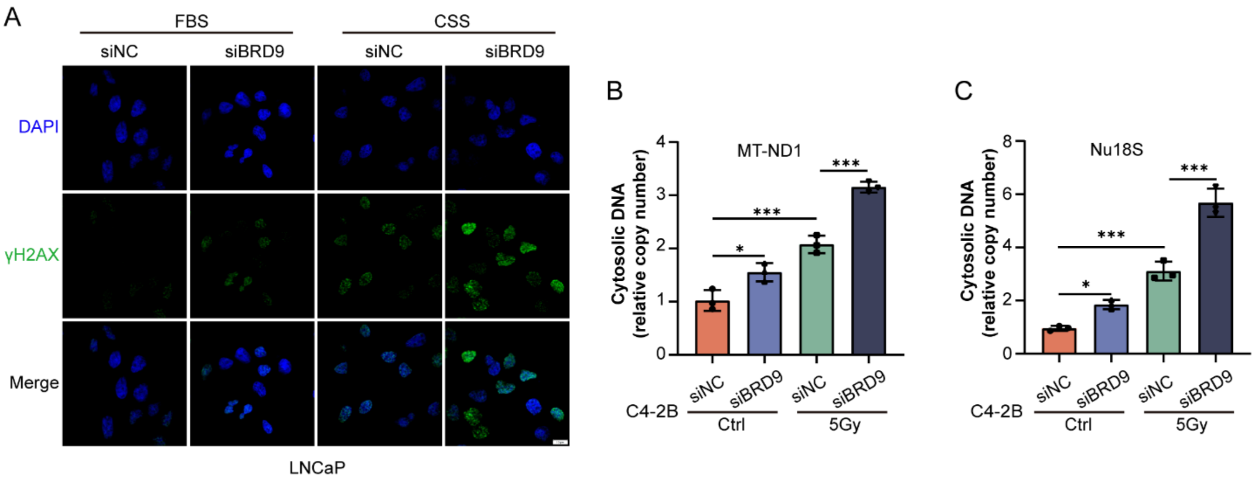

Fig.S9 BRD9 knockdown induces DNA damage.

A Immunofluorescence staining assay of DNA double-stranded breaks (DSBs) in LNCaP cells with BRD9 knockdown with or without androgen deprivation (n = 3). Scale bars, 10 μm. **B, C** Relative levels of mitochondrial (MT-ND1) and nuclear (Nu18S) DNA in C4-2B cells with or without BRD9 silencing (siBRD9 or siNC, respectively) in response to X-ray irradiation (n = 3). Gy, Gray. FBS, fatal bovine serum. CSS, charcoal-stripped serum. One-way analysis of variance (ANOVA). Error bars represent SD; **P* < 0.05, ***P* < 0.01, *** *P* < 0.001.

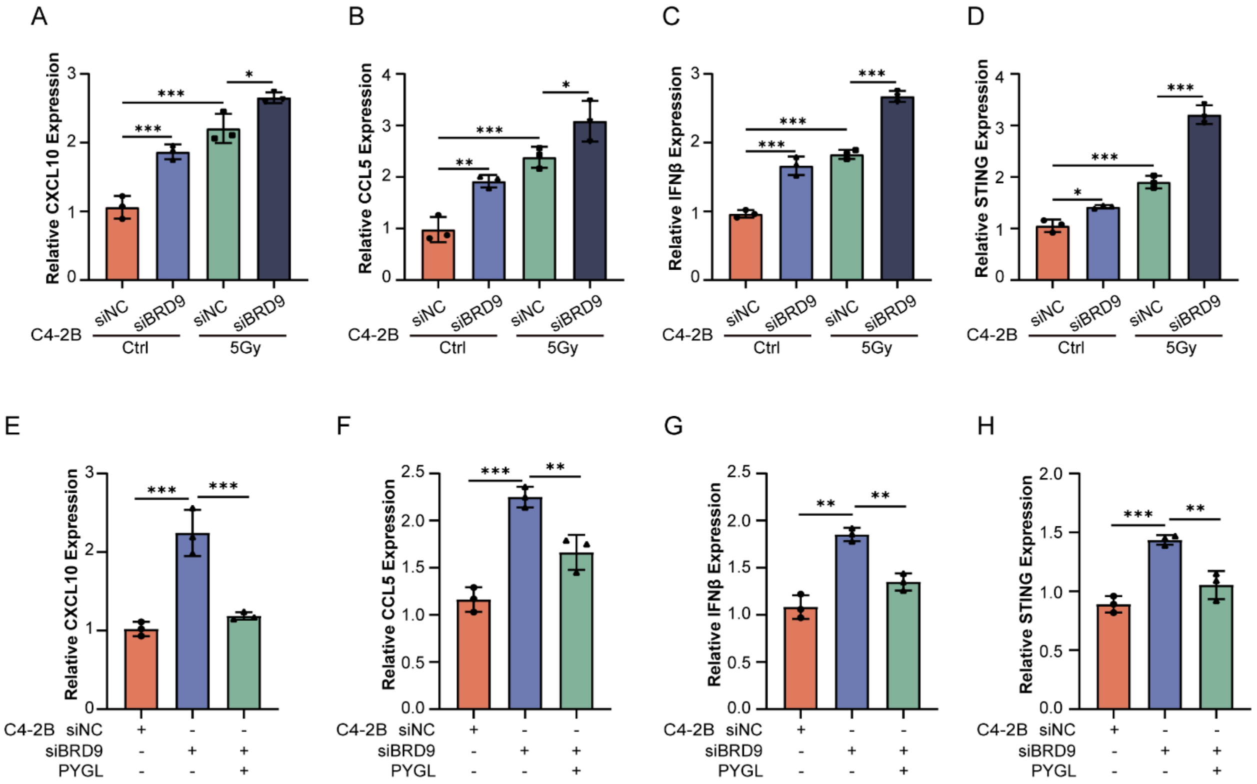

Fig.S10 BRD9 regulates the activity of the cGAS-STING signaling pathway in a PYGL-dependent way.

A-D The relative mRNA levels of CXCL10, CCL5, INFβ, and STING in C4-2B cells with or without BRD9 silencing (siBRD9 or siNC, respectively) in response to X-ray irradiation (n = 3). Gy, Gray. **E-H** The relative mRNA levels of CXCL10, CCL5, INFβ, and STING in C4-2B cells were transfected with siBRD9 or siNC for 24 hours and subsequent addition of the PYGL overexpression plasmid for another 24 hours. Gy, Gray. One-way analysis of variance (ANOVA). Error bars represent SD; **P* < 0.05, ***P* < 0.01, *** *P* < 0.001.

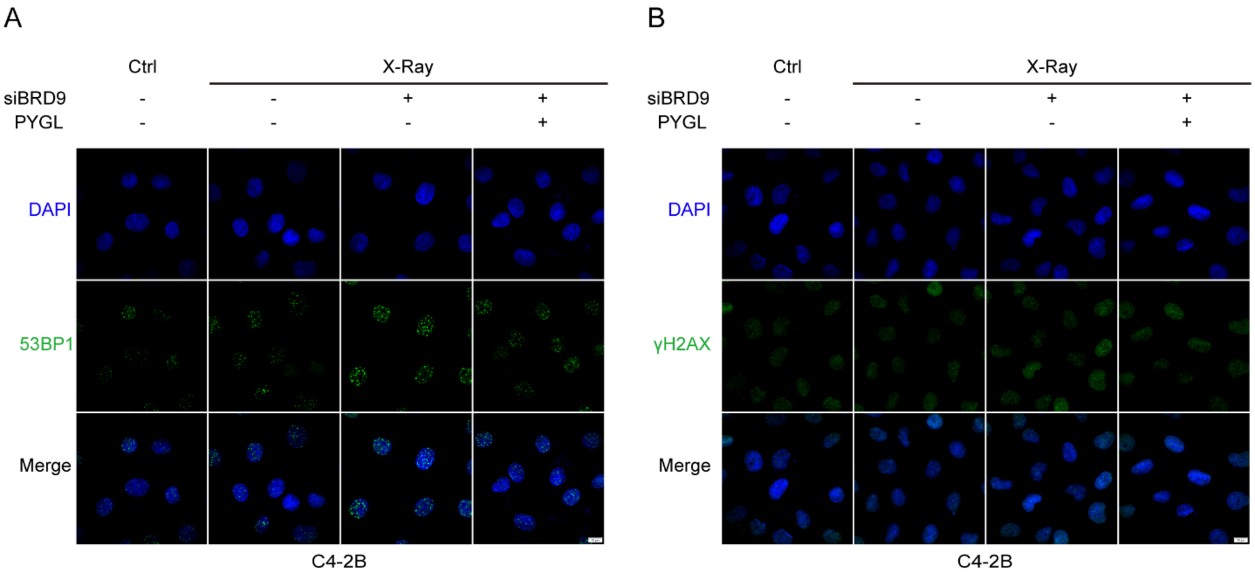

Fig.S11 BRD9 provokes DNA damage in a PYGL dependent-way.

A-B Immunofluorescence staining assay of DNA double-strand breaks (DSBs) markers 53BP1 and γH2AX in C4-2B cells co-transfected with siBRD9 for 24 hours and then with the PYGL overexpression plasmid for another 24 hours under challenge with X-ray irradiation (n = 3). Scale bars, 10 μm.

Supplemental Materials and Methods

Comet assay

Single cell gel electrophoresis kit (Abbkine, KTA3040) was used for comet assay. The transfected cells were fixed on Comet slides with low melting point agarose and lysed in the dark at 4 °C for 60min, followed by replacement with precooled alkaline despiralization solution (approximately 25 mL/Comet Slide) at 4 °C for 30 min. Following electrophoresis in 35 V alkaline electrophoresis buffer (1mM EDTA, 300mM NaOH, PH > 13) for 30 min, gels were then neutralized with Tris–HCl buffer (0.4 mM, PH = 7.5, 3 times, 10 min) and stained with PI. Cells were photographed using fluorescence microscopy, and comet tails were analyzed by ImageJ software.

Reduced glutathione (GSH) and oxidized glutathione (GSSG) assay

The GSH and GSSG levels were determined with the GSH and GSSG Detection Kit (BC1175, BC1185, Solarbio, Beijing, China). The 1×10^6 PCa cells were subjected to sonication in ice to ensure efficient disruption. Subsequently, the sonicated cell suspension was centrifuged at 12,000g, 4°C for 10 minutes. The supernatant was carefully collected for further measurement. Finally, GSH and GSSG were measured at 412 nm by the SpectraMax iD3 Multi-Mode Microplate Reader (Molecular Devices, US) and the ratios (GSH/GSSG) were calculated to determine the redox state.

Mitochondrial membrane potential (MMP) analysis

Mitochondrial membrane potential assay kit with 5,5′,6,6′-tetrachloro-1,1′,3,3′-tetraethyl-imidacarbocyanine iodide (JC-1) (C2006) was bought from Beyotime. JC-1 is a cationic, positively charged fluorescent dye that shows potential dependent

accumulation in mitochondria. The fluorescence intensity of JC-1 monomers (Ex.490 nm, Em.530 nm) and aggregates (Ex.525 nm, Em.590 nm) was measured using the SpectraMax iD3 multimode enzyme-labeled apparatus (Molecular Devices, US). MMP was measured as the ratio of JC-1 aggregates to monomers.

Measurements of glucose consumption, lactate production and G6PD activity

Glucose and lactate levels in PCa cells were measured by the glucose colorimetric assay kit (S0201S, Beyotime Biotechnology, Jiangsu, China) and lactate assay kit (KTB1100, Abbkine Scientific, Wuhan, China), respectively. G6PD enzyme activity was measured using the G6PD assay kit (S0189, Beyotime Biotechnology, Jiangsu, China). The assay outputs were measured using a SpectraMax iD3 multimode microplate reader (Molecular Devices, US).

Glutathionylation assay

Intracellular glutathione-modified proteins were labeled by streptavidin-HRP for 4 hours post BioGEE (250μM) (a cell-permeant, biotinylated glutathione analog for detection of S-glutathionylation) treatment. After immunoprecipitation with streptavidin, the fragments were cleaved by nonreducing SDS-PAGE and blotted with anti-BRD9 antibody.

Transfection with wild-type Flag-BRD9 vector (WT), or vectors expressing Flag-BRD9 with mutations of C101 (C101S), C129 (C129S), C209 (C209S), C279(C279S), C288 (C288S) or C421 (C421S), immunoprecipitation was performed with anti-FLAG antibody, and glutathionylation was detected with anti-GSH antibody. Dithiothreitol (DTT), a reducing reagent, was used for the control.

ROS assay

Intracellular ROS levels were assessed using 2',7'-dichlorofluorescein diacetate (DCFH-DA; BestBio, BB-4705-2). The ROS probe was diluted at a ratio of 1:1,000 with fresh serum-free medium. After thorough mixing, the diluted probe was stored in the dark until use. The cells were incubated with DCFH-DA at 37°C for 20 min and then washed three times in serum-free medium to measure fluorescence (Ex.488 nm, Em.525 nm) by the SpectraMax iD3 Multi-Mode Microplate Reader (Molecular Devices, US). The measured data were standardized with control.

DHE-ROS assay kit (BB-47051, BestBio, Shanghai, China) was used according to the standard protocol to detect the ROS levels. The tissue was frozen in liquid nitrogen to -80°C, then the frozen tissue was cut into 5μm thick sections, autofluorescence quench was added, ROS was dropped for staining, sections were rinsed, and finally sealed. Photographs were taken using a fluorescence microscope.

NADPH/NADP$^+$ levels assay

The 1 × 10^6 PCa cells were added 200ul of lysate and centrifuged at 12,000g, 4°C for 10 minutes. Intracellular levels of NADPH and NADP$^+$ were determined by using the NADP/NADPH-GLO kit (Promega, Madison, Wisconsin, USA) according to the manufacturer's instructions. The ratios (NADPH/NADP$^+$) were calculated to determine the redox state.

Chromatin immunoprecipitation

Chromatin immunoprecipitation (ChIP) was carried out using the ChIP assay kit (Beyotime Biotechnology, Jiangsu, China) following the manufacturer's instructions.

Briefly, chromatin from pretreated cells was fixed with 1% formaldehyde for 10 min at room temperature. DNA was broken into 200–1000 bp fragments using a sonicator (10^6 cells in 250 μL volume; ultrasound for 15s, pause for 15s, repeated eight times). Chromatin was immunoprecipitated with anti-BRD9 (Abcam, Cambridge, MA, USA) and control IgG (St. Louis, MO, USA) antibodies. The association of BRD9 with PYGL promoter was measured by qRT-PCR (pre-denaturation at 95°C for 5 min; followed by 95°C for 30 s, 65°C for 30 s, and 72°C for 30 s, 35 cycles) using immunoprecipitated chromatin from cells with the indicated primers listed in Supplementary Table 1.

Measurement of mitochondrial respiration and glycolytic capacity

An extracellular flow analyzer (XF96: Seahorse Biosciences Agilent, Santa Clara, CA, USA) was used to analyze mitochondrial function as previously described [31]. In brief, LNCaP or C4-2B cells were seeded in each well of a Seahorse XFe96 cell culture plate. Oxygen consumption rate (OCR) was measured under basal conditions and after addition of oligomycin (1 μM), FCCP (1 μM), rotenone and antimycin (0.5 μM). In addition, cells from each group were cultured in conditioned medium and in the absence of CO_2 for 1 h and calibrated. After injection of glucose, oligomycin, and 2-DG (Sigma), the extracellular acidification rate (ECAR) of cells in each group was measured. Data were normalized according to cell density.

Mitochondrial morphology analysis

C4-2B cells were transfected with siBRD9 or siNC for 24 hours. Then cells were washed, harvested, and fixed at 4°C for 24 hours with Fixing Solution (G1102, Servicebio). The cells were then post-fixed in 1% osmium tetroxide, dehydrated in a

graded series of ethanol, infiltrated, and embedded in EMBed. Ultrathin sections were evaluated using a HT7700 transmission electron microscope (HITACHI).

Supplementary Table 1: Primer sequences

RT-qPCR		
**Gene name**	**Forward (5'-3')**	**Reverse (5'-3')**
BRD9	GCCACGACTCCAGTTACTATG	TCTCCTTCTCGGACTTCTTCT
PYGL	CACTTCAGTGGCAGATGTGGTG	GCAGTGGAAATCTGCTCTGACAG
STING	GCTGCTGTCCATCTATTTCTACT	GCCGCAGATATCCGATGTAATA
IFNβ	TTGTTGAGAACCTCCTGGCT	TGACTATGGTCCAGGCACAG
CCL5	CGCTGTCATCCTCATTGCTA	CCAGACTTGCTGTCCCTCTC
CXCL10	CTGTACGCTGTACCTGCATCA	TTCTTGATGGCCTTCGATTC
HK2	GACCAACTTCCGTGTGCTTT	TCCATGAAGTTAGCCAGGCA
PKM2	ATGGCTGACACATTCCTGGA	AGAAGTTCAGACGAGCCACA
LDHA	CAACATGGCAGCCTTTTCCT	ACCCACCCATGACAGCTTAA
G6PD	TCAACAGCCACATGAATGCC	ACAGGGAGGAGATGTGGTTG
α-tubulin	TACGGAAAGAAGTCCAAGC	CTGAGGGAAGCAGTGATG
Nu18S	TAGAGGGACAAGTGGCGTTC	CGCTGAGCCAGTCAGTGT
mtND1	CACCCAAGAACAGGGTTTGT	TGGCCATGGGTATGTTGTTAA
GAPDH	CGGAGTCAACGGATTTGGTCGTAT	AGCCTTCTCCATGGTGGTGAAGAC
GCLC	CTGCATCTGTAGATGATAGAACTC	CAGCTGTTCGTAGATCTCTTTATC
GPX4	ATCCTGGGAAATGCCATCAAG	AAATAGTGGGGCAGGTCCTTC
SLC7A11	GCTTTGTCTTATGCTGAATTGG	TGCAGGGCGTATTATGAGGAG
PRDX3	CGACATGTGAGTGCCATTCC	AGCAGGTGCATGGCATGAG
SOD1	GGTCCTCACTTTAATCCTCTATCCAG	CCAACATGCCTCTCTTCATCC
**ChIP-qPCR**		
**PYGL**	**Forward primer**	**Reverse primer**
P1	CACTCTGCCTGGGTAACA	CTATCTCCTGACCTCGTGAT
P2	ACAGAGTGAGACTCCGTC	AACAGTCCATTGCCAGAAT
P3	GCACTTCCTCTGCCCTCC	TTTCAACGCGCACGAAAGTT

Supplementary Table 2: Antibodies

Antibodies	Source	Identifier
Rabbit anti-Androgen Receptor antibody	Cell Signaling Technology	Cat# 5153
Rabbit anti-BRD9 antibody	Cell Signaling Technology	Cat# 58906
Rabbit anti-53BP1 antibody	Cell Signaling Technology	Cat# 4937S
Mouse anti-P-Histone H2A.X antibody	Cell Signaling Technology	Cat# 80312
Rabbit anti-IRF3 antibody	Cell Signaling Technology	Cat# 4302
Rabbit anti-P-IRF3 antibody	Cell Signaling Technology	Cat# 29047
Rabbit anti-TBK1 antibody	Cell Signaling Technology	Cat# 3013
Rabbit anti-P-TBK1 antibody	Cell Signaling Technology	Cat# 5483
Rabbit anti-PYGL antibody	Proteintech	Cat# 15851-1-AP
Mouse anti-GAPDH antibody	Proteintech	Cat# 60004-1-Ig
Mouse anti-GSH antibody	Virogen	Cat# 101-A
Rabbit anti-FLAG antibody	Abcam	Cat# ab205606
Mouse anti-NFYA antibody	Santa Cruz Biotechnology	Cat# sc-17753
Mouse anti-BRG1 antibody	Santa Cruz Biotechnology	Cat# sc-17796
Streptavidin-HRP	Beyotime	Cat# A0303

Supplementary Table 3: siRNA sequences

siRNA	Target sequence
BRD9	GGACGCUCUUCCAGCUGAATT
	GCCUGCAGCUUGACGGACATT
PYGL	CCUGUGAUGAGGCCAUUUATT
	CCCGGCUACAUGAAUAACATT
NFYA	GGCCAGCUAAUCACAUCAATT
	CGUCUAUCAACCAGUUAAUTT

Original western blots

Fig1B

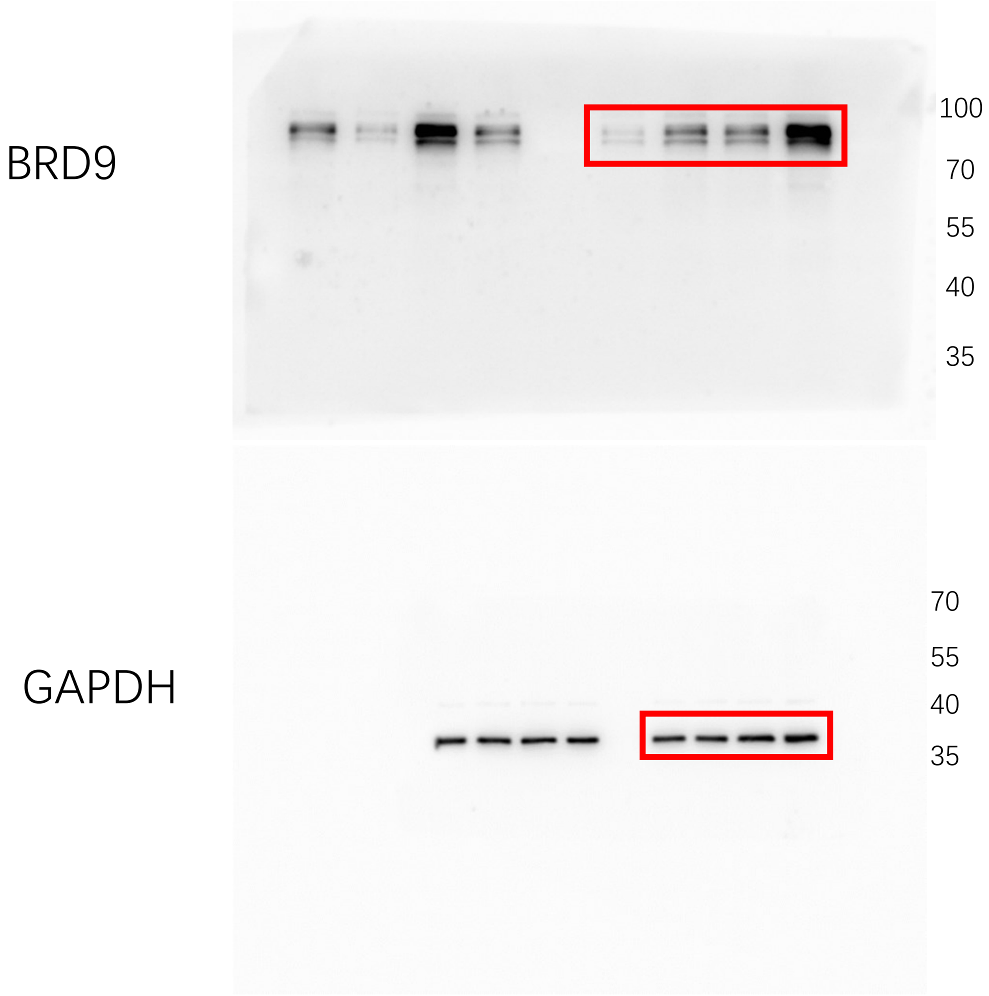

Fig3B

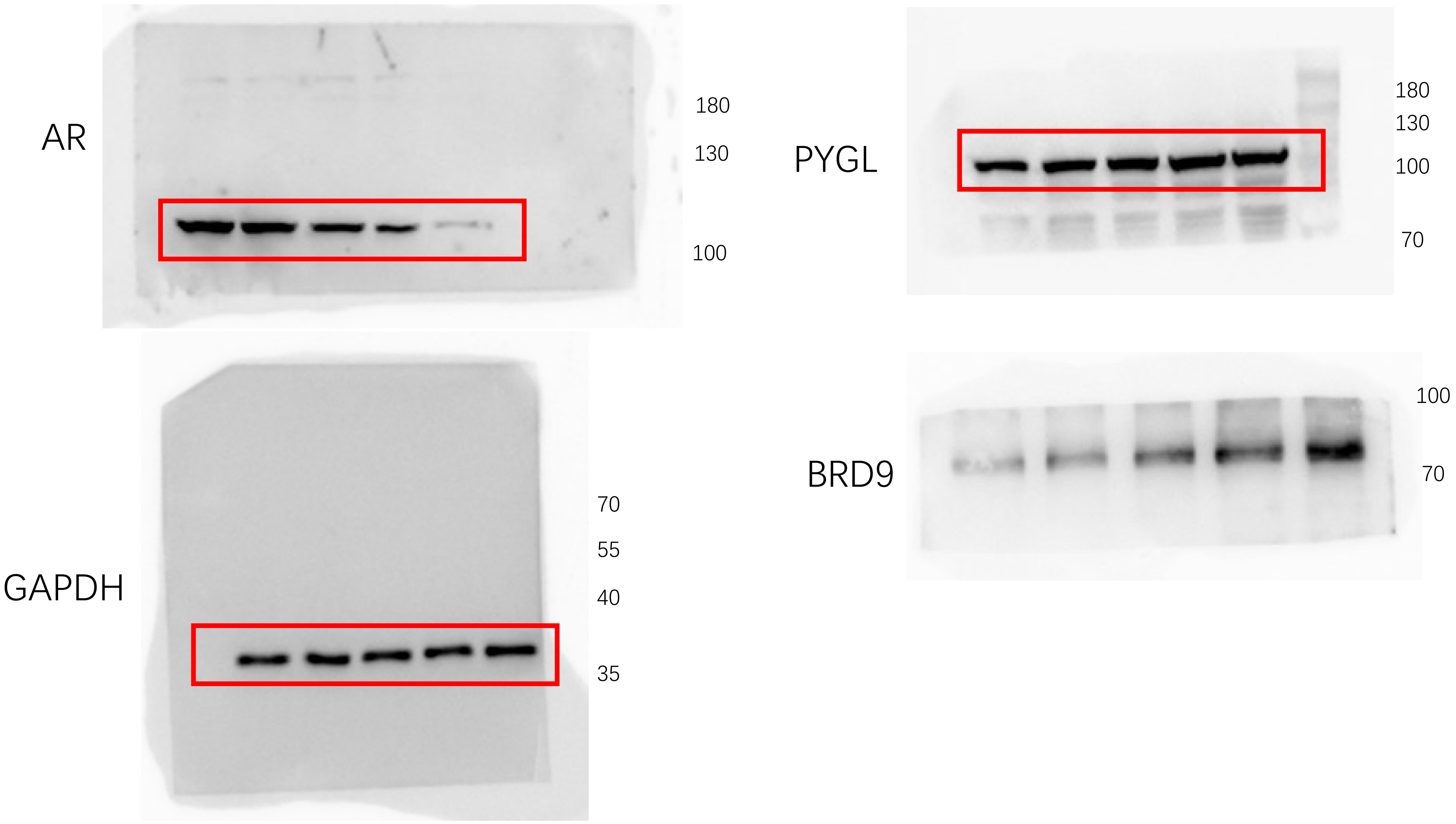

Fig5A

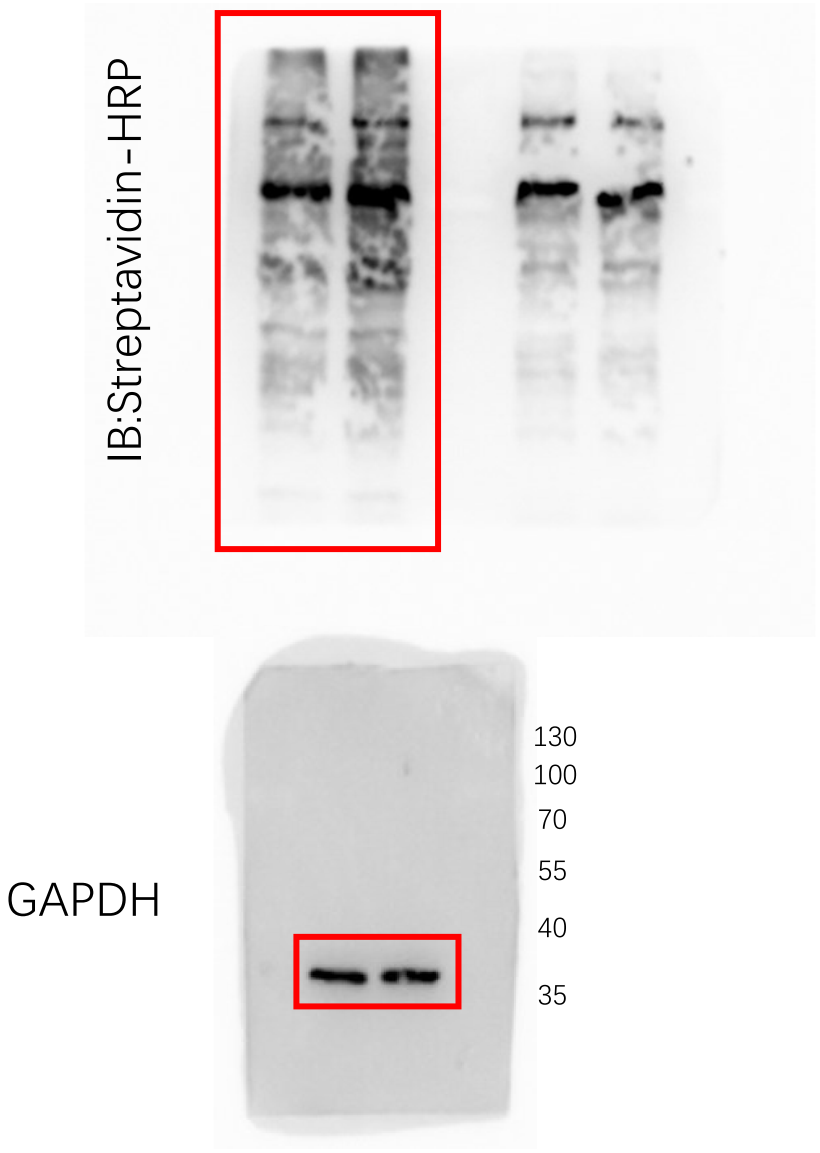
IB:Streptavidin-HRP
GAPDH
130
100
70
55
40
35

Fig5C

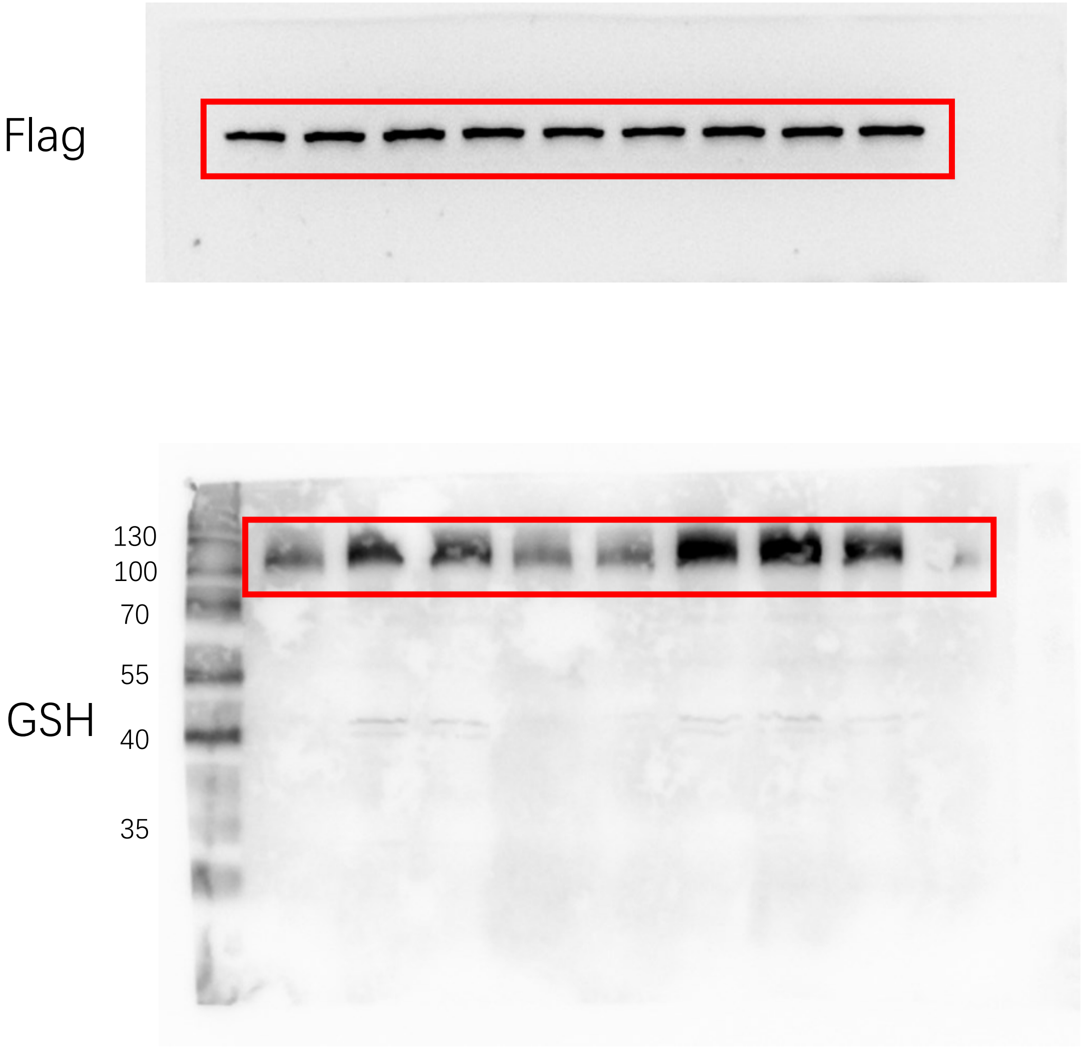
Flag
GSH
130
100
70
55
40
35

Fig6E

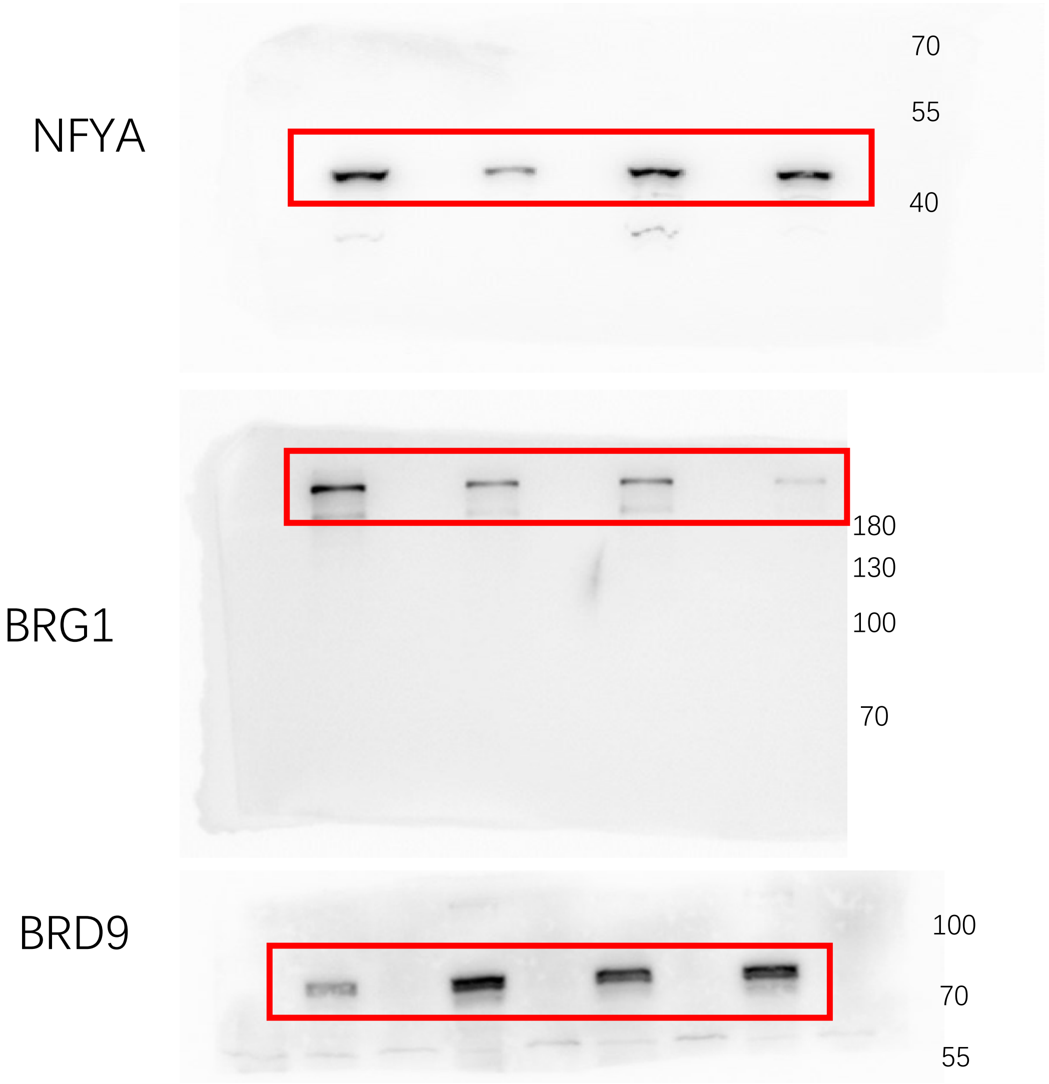

Fig7E

γH2AX

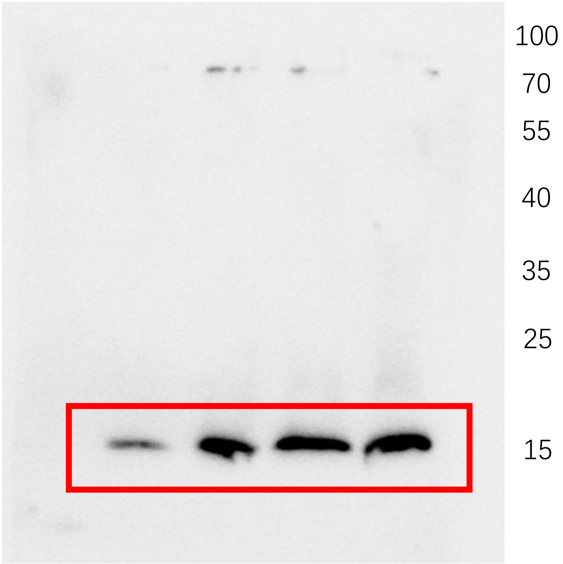

BRD9

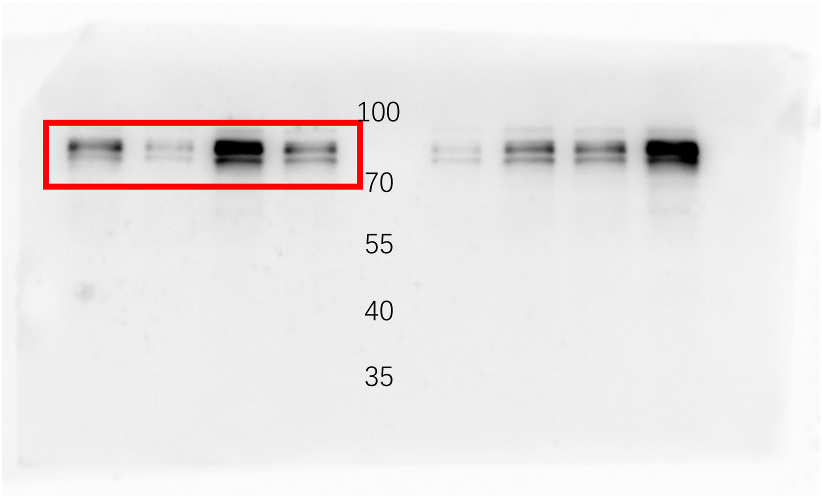

GAPDH

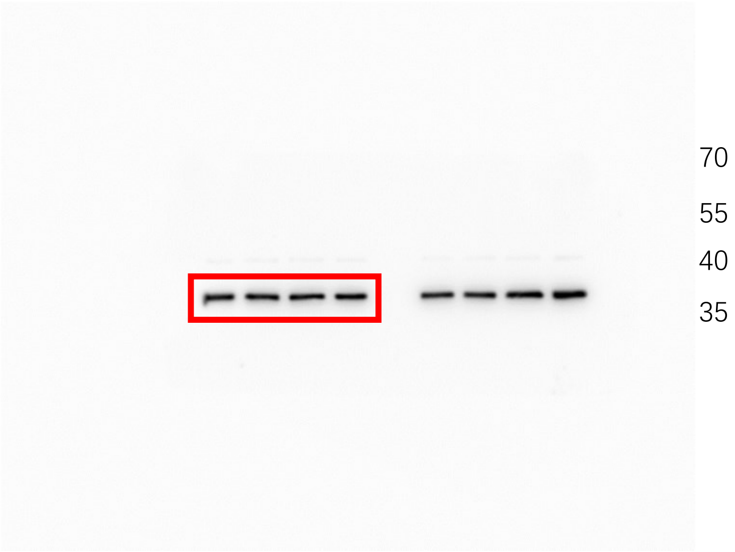

Fig7F

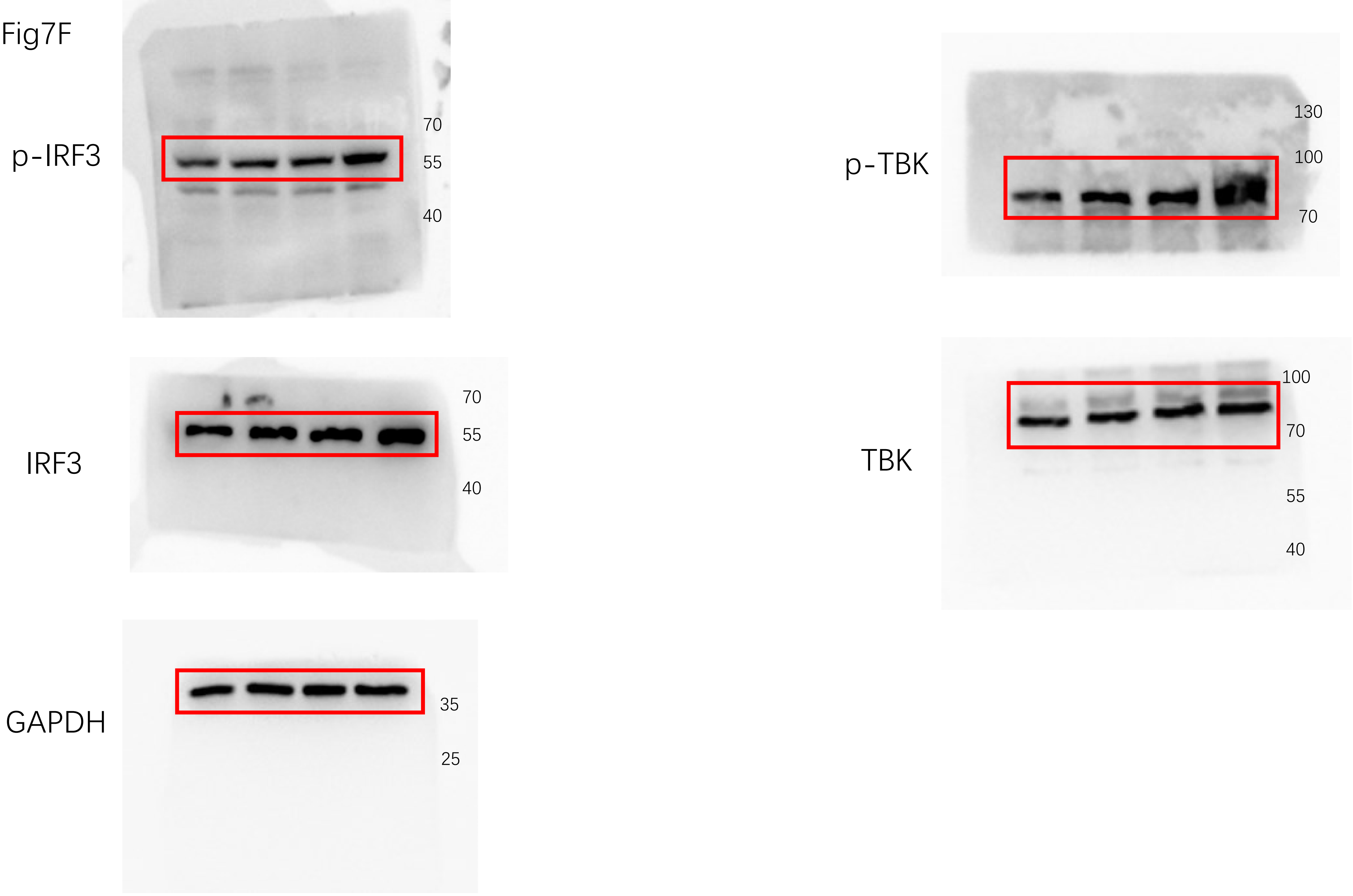
p-IRF3
70
55
40
IRF3
70
55
40
GAPDH
35
25
p-TBK
130
100
70
TBK
100
70
55
40

FigS8

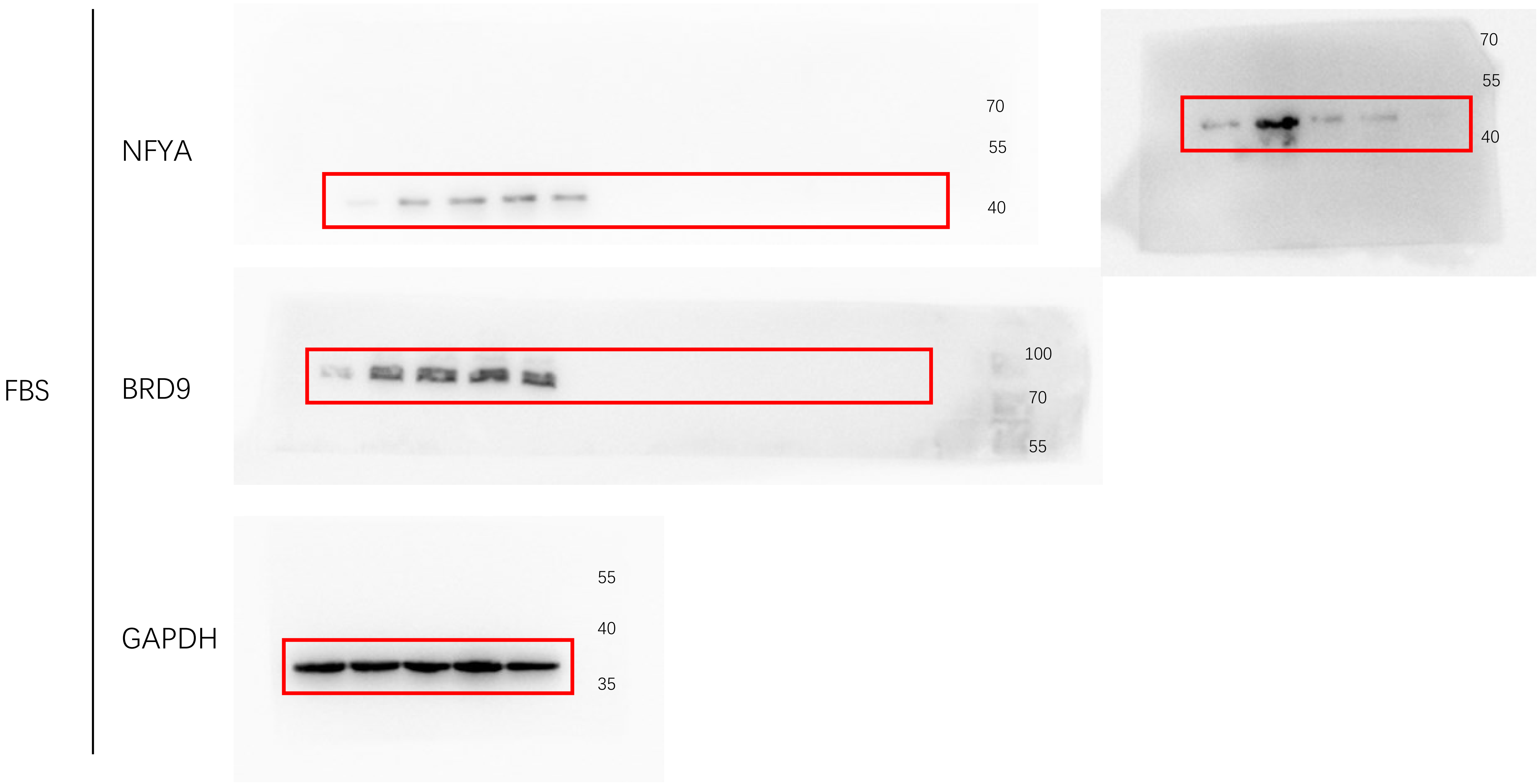

FigS8

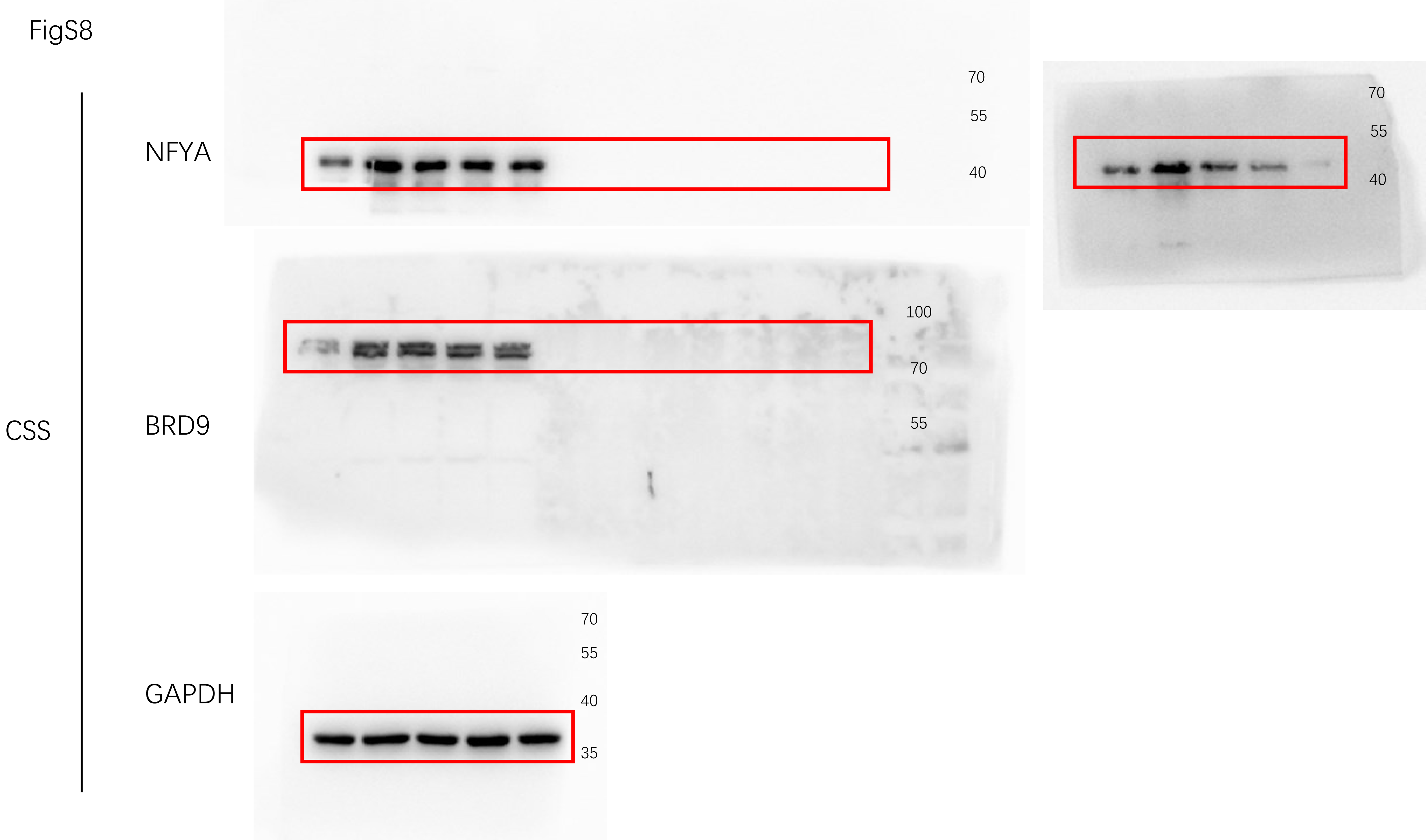

Supplement: Supplementary file 1 — Supplementary materials [file 41419_2025_7561_MOESM1_ESM.pdf]
